# Genomic and transcriptomic analyses reveal adaptation mechanisms of an *Acidithiobacillus ferrivorans* strain YL15 to alpine acid mine drainage

**DOI:** 10.1371/journal.pone.0178008

**Published:** 2017-05-19

**Authors:** Tangjian Peng, Liyuan Ma, Xue Feng, Jiemeng Tao, Meihua Nan, Yuandong Liu, Jiaokun Li, Li Shen, Xueling Wu, Runlan Yu, Xueduan Liu, Guanzhou Qiu, Weimin Zeng

**Affiliations:** 1 School of Minerals Processing and Bioengineering, Central South University, Changsha, China; 2 Key Laboratory of Biometallurgy, Ministry of Education, Central South University, Changsha, China; 3 School of Metallurgy and Environment, Central South University, Changsha, China; 4 CSIRO Process Science and Engineering, Clayton, Victoria, Australia; National Renewable Energy Laboratory, UNITED STATES

## Abstract

*Acidithiobacillus ferrivorans* is an acidophile that often occurs in low temperature acid mine drainage, e.g., that located at high altitude. Being able to inhabit the extreme environment, the bacterium must possess strategies to copy with the survival stress. Nonetheless, information on the strategies is in demand. Here, genomic and transcriptomic assays were performed to illuminate the adaptation mechanisms of an *A*. *ferrivorans* strain YL15, to the alpine acid mine drainage environment in Yulong copper mine in southwest China. Genomic analysis revealed that strain has a gene repertoire for metal-resistance, e.g., genes coding for the *mer* operon and a variety of transporters/efflux proteins, and for low pH adaptation, such as genes for hopanoid-synthesis and the sodium:proton antiporter. Genes for various DNA repair enzymes and synthesis of UV-absorbing mycosporine-like amino acids precursor indicated hypothetical UV radiation—resistance mechanisms in strain YL15. In addition, it has two types of the acquired immune system–type III-B and type I-F CRISPR/Cas modules against invasion of foreign genetic elements. RNA-seq based analysis uncovered that strain YL15 uses a set of mechanisms to adapt to low temperature. Genes involved in protein synthesis, transmembrane transport, energy metabolism and chemotaxis showed increased levels of RNA transcripts. Furthermore, a bacterioferritin Dps gene had higher RNA transcript counts at 6°C, possibly implicated in protecting DNA against oxidative stress at low temperature. The study represents the first to comprehensively unveil the adaptation mechanisms of an acidophilic bacterium to the acid mine drainage in alpine regions.

## Introduction

Acid mine drainage as a typical extreme environment is associated with metal or coal mines and derelict mines. It is often highly acidic (typically pH<3) and usually contain elevated concentrations of zinc, copper and a variety of other heavy metals [1]. Acid mine drainage is common on the earth. It is distributed among mine sites with distinct climatic conditions, even those mainly characterized by low temperatures. For instance, a number of mine sites are located at high altitudes and latitudes, of which the temperatures are below 10°C for the majority of the year [2,3].

The harsh conditions in low temperature acid mine drainage inhibit growth of most organisms. Despite the harshness, some microorganisms can survive in this extreme environment, one of which is the gammaproteobacterium *Acidithiobacillus ferrivorans* [4]. The bacterium accounts for a considerable part in cold mine-affected water bodies. The species can grow at subzero temperatures and has a fastest growth temperature around 30°C. According to a recent conception, all microorganisms that are indigenous to cold environments are psychrophiles [5]. Therefore, the species *A*. *ferrivorans* should be psychrophilic rather than psychrotolerant as it was formerly considered to be [4].The species is an iron- and sulfur-oxidizing, diazotrophic, obligate chemoautotroph. It was once regarded as cold-adapted *A*. *ferrooxidans*, however, it differs from *A*. *ferrooxidans* in their cell motility, tolerance to low temperature and responses to pH [4]. Apart from the physiological aspects, its ferrous and reduced inorganic sulfur compounds oxidation pathways have been well discussed [6,7]. The species is implicated in biomining for the recovery of metals from sulfide minerals at low temperatures and this has been studied [8]. Christel et al. [9] found that when using potassium tetrathionate as an energy source, the *A*. *ferrivorans* strain SS3 had little RNA transcript response related to cold stress and thus it was concluded that the strain is adapted to growth at 8^°^C.

Though there are some studies on the microbial adaptation mechanisms to acid mine drainage, e.g. reports by Hua et al. [10] and Liljeqvist et al. [11], few has been conducted on microorganisms in acid mine drainage environments in alpine regions at a strain-level, e.g. those located in High Andes in Chile [3] and Tibet Plateau in China, which is characterized by low temperature and elevated UV radiation (UVR). Microbial adaptation to environment associates with a variety of biological processes. A large number of genes are implicated in these processes. Therefore, it is difficult to barely explain the mechanisms by traditional culture-dependent or modern molecular biological approaches. The advent of high-throughput next-generation sequencing (NGS) technology now affords new opportunities to address the knowledge gaps by comprehensively characterizing the genes and processes that are involved in the adaptation by microorganisms to their habitats. For instance, comparative transcriptomic analysis reveals the adaptation of microbial communities to acid mine drainage in south China [12]. We presented here the adaptation-related gene repertoire of an *A*. *ferrivorans* strain YL15 isolated from acid mine drainage in an alpine copper mine, using genomic and transcriptomic methods. In particular, we focused on the potential strategies the strain uses to cope with the abiotic and biotic constraints of its natural habitats including extremely acidic pH, high metal ion concentrations, UVR, low temperature and intrusion of extraneous genetic elements.

## Materials and methods

### Strain and culture conditions

Strain YL15 was isolated from acid mine drainage in Yulong copper mine in Tibet, China. The sampling site was located at an altitude of about 4,600 meters (31°21’34'‘N, 97°46’57”E), and the physicochemical properties of the acid mine drainage was listed in S1 Table. No specific permissions were required for activities in Yulong copper mine, because the mine is available to the public. The sampling did not have any impact on the local environment and this field study did not involve endangered or protected species. The strain was isolated using FeTSB solid medium as described previously [13]. It has a fastest growth at 28°C when ferrous sulfate is used as an energy source. It was grown routinely at pH of 2.0 and temperatures of 28°C and 6°C in shake flasks at 160 rpm. In this study, we selected 6°C as the low temperature because it is a typical temperature occurred in Yulong copper mine. The culture medium was 9K [14] and filtration sterilized ferrous sulfate was supplied at a concentration of 50 mM.

### Nucleic acid extraction

For DNA extraction, strain was cultured at 28°C until it entered the mid logarithmic phase. Bacterial cells were harvested by centrifugation at 10,000x g for 10 min. The pelleted cells were washed twice using diluted sulphuric acid (pH 2.0). Genomic DNA was extracted and purified from the washed cells using TIANamp Bacteria DNA kit (TIANGEN, Beijing, China) as per the manufacturer’s instructions and finally suspended in TE buffer. The genomic DNA was quantified by ethidium bromide-UV detection on an agarose gel and stored in -80°C until used for genome sequencing.

As for RNA extraction, strain was respectively cultured at 28°C and 6°C (designated as S28 and S6). When cells entered the mid logarithmic phase at 42h (for S28) and 144h (for S6) respectively (S1 Fig), the cultures were rapidly cooled and harvested by centrifugation at 10,000x g for 5 min at 4°C. Total RNA was extracted by using Total RNApure kit (Zoman, Beijing, China) according to the manufacturer’s instructions. Trace genomic DNA was digested using DNase I. The quality of total extracted RNA was confirmed by 1% agarose gel electrophoresis and quantified using a NanoDrop ND-1000 Spectrophotometer (NanoDrop Technologies, Wilmington, USA).

### Genome sequencing, assembly and annotation

The purified genomic DNA sample was used to construct a shotgun library with an average insert size of ~300 bp. The tagging and fragmentation of genomic sample, indexing and PCR amplification, PCR clean-up, library normalization and pooling were conducted using the Illumina Nextera XT DNA Sample Preparation Kit (Illumina, California, USA) as per the manufacturer’s instructions. The library was then sequenced (250 bp paired-end reads) using the Illumina MiSeq sequencing platform (Illumina, California, USA). The raw reads were assembled into contigs using SOAPdenovo2 package [15]. The genome completeness was estimated using the program CheckM [16]. Coding sequences were predicted with the ORF finders Glimmer [17] and GeneMark [18]. All CDSs were manually verified by alignment against the NCBI non-redundant [19] and COG databases [20] using the BLAST software [21]. In particular, the *cusCBA* gene clusters were identified by method described by González et al [22]. Clustered regularly interspaced short palindromic repeats (CRISPRs) loci were identified using the web server CRISPRFinder [23]. The tRNA and rRNA genes were identified using the software tRNAscan-SE [24] and the webserver RNAmmer [25], respectively. This Whole Genome Shotgun project has been deposited at DDBJ/ENA/GenBank under the accession MASQ00000000. The version described in this paper is version MASQ01000000.

### Genome homology and synteny analyses

The average nucleotide identity (ANI) between genomes of strain YL15 and *A*. *ferrooxidans* ATCC 23270, *A*. *thiooxidans* ATCC 19377, and the other two *A*. *ferrivorans* strains SS3 [26] and CF27 [27], was calculated using OrthoANI [28]. Genome synteny analysis between genomes of strain YL15 and *A*. *ferrivorans* strain SS3 and *A*. *ferrooxidans* ATCC 23270 was performed using the Nucmer program in the MUMmer package using the default parameters [29].

### RNA-seq and analysis of genes with significantly different RNA transcript counts

For sequencing of YL15 mRNA, rRNA was removed from total RNA using the Ribo-Zero Magnetic Kit (Bacteria, Epicentre Biotechnologies, Wisconsin, USA), then the remaining RNA was employed for library construction using the Illumina TruSeq RNA Sample Preparation Kit (Illumina, California, USA) according to the manufacturer’s instructions. Briefly, mRNA was fragmented into small pieces using divalent cations under elevated temperature. The cleaved RNA fragments were then copied into first strand cDNA using random primers and reverse transcriptase. Second strand cDNA synthesis followed, using RNase H and DNA polymerase. The cDNA fragments then went through an end repair process, the addition of a single ‘A’ base to 3’ ends, and then ligation of the adapters. The products are then purified and enriched with PCR to create the final cDNA library. The library was then sequenced (75 bp paired-end reads) using the Illumina MiSeq sequencing platform (Illumina, California, USA). The raw sequencing data was deposited at Sequence Read Archive under the accession SRP091325.

Clean data were obtained from raw data by removing reads that containing low quality reads, adapter, and poly-N. Computational processing and analysis of qualified reads were conducted through the pipeline using TopHat and Cufflinks packages for identification of genes with significantly different RNA transcript counts [30]. The qualified reads for each condition were mapped to the YL15 genome with TopHat. The resulting alignment files were provided to Cufflinks to generate a transcriptome assembly for each condition. Cuffmerge program, which is included in the Cufflinks package, was used to merged together the assemblies. Cuffdiff, another utility in the Cufflinks package, calculated transcript expression levels, performed differential analysis and tested the statistical significance of observed changes (*p*-value). Significantly differentially expressed genes were determined with a selection threshold of adjusted *p*-value ≤ 0.05 and fold change of RNA transcripts ≥ 2.0 (up-regulation) or ≤ 0.5 (down-regulation). Gene ontology (GO) was implemented using TBtools (https://github.com/CJ-Chen/TBtools) and WEGO [31] and KOBAS 2.0 was employed to conduct KEGG pathway mapping analysis against the KEGG background [32].

### Quantitative real-time PCR (qRT-PCR) to verify RNA-seq data

The extracted RNA was first retro-transcribed into cDNA with Reverse Transcriptase (Zoman, Beijing, China) and random primers following the manufacturer’s instructions. Real-time PCR was carried out with the iCycler iQ Real-time PCR detection system (Bio-Rad Laboratories, USA) as previously reported [33]. Primers for selected genes were listed in S2 Table. The absolute quantification of each gene was carried out by making standard curves. All tests were carried out in triplicate.

## Results and discussion

### Genomic analysis of strain YL15

#### Genomic features

The draft genome sequence of strain YL15 has a total length of 2,996,582 bp, with a GC content of 56.6%. After assembly, a total of 190 contigs was created, ranging from 200 bp to 105,515 bp. Given a 99.03% genome completeness provided by CheckM, and a 123x genome coverage, it is reasonable to infer that the majority of genes in genome of strain YL15 were included in the current draft. Comparing to the other two *A*. *ferrivorans* strain SS3 and CF27, the genome of YL15 is smaller, and in particular, 12.7% shorter than the genome of CF27 in length (https://www.ncbi.nlm.nih.gov/genome). The genome has 43 tRNA genes and 2,798 protein-coding sequences, of which 1,852 were assigned as proteins with known functions, while the rest 946 were regarded as hypothetical proteins.

**Fig 1:**
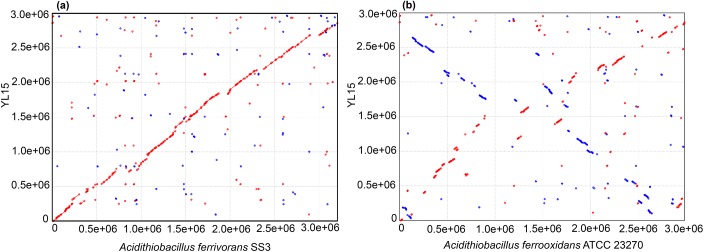
Dot plots for synteny of YL15, *A*. *ferrivorans* SS3 and *A*. *ferrooxidans* ATCC 23270 genomes. In the plots, every dot indicates a match between the two genomes being compared, with forward matches colored in red and reverse matches colored in blue.

The genome has one rRNA operon, and the similarities of 16S rDNA to those of other *A*. *ferrivorans* strains are over 99%. The ANI values calculated by OrthoANI for strains YL15 and SS3, CF27, *A*. *ferrooxidans* ATCC 23270 and *A*. *thiooxidans* ATCC 19377 were 96.90%, 98.42%, 84.03% and 73.84%, respectively. According to the ANI threshold of 95% which has been proposed for species demarcation, YL15 is affiliated to *A*. *ferrivorans* [34]. The results were consistent with that of synteny analysis. Genome of strain YL15 has a high degree of synteny with that of strain SS3, while few synteny regions were observed between the YL15 genome and that of *A*. *ferrooxidans* ATCC 23270 (Fig 1).

#### Metal resistance

Elevated concentrations of metal ions especially heavy metals are toxic to microbial cells, mainly as a result of their ability to denature protein molecules [35]. In response to the toxic assault, microorganisms have developed a set of resistance mechanisms. In general, these mechanisms include: (i) converting the ions to less toxic forms and then pump them out of cells; (ii) exporting the metal ions to the periplasm and reduce them to lower oxidative or decreased soluble states; (3) exporting the ions out of the cell entirely [36].

A large number of genes that are predicted to be involved in metal resistance were identified in genome of strain YL15, including: i) *mer* operon for mercuric resistance and regulation (S3 Table); ii) genes for arsenic resistance: an arsenate reductase (BBC27_RS06310), an ATP-dependent chaperone gene *clpB* (BBC27_RS08660) and an *arsRCDA* operon (S3 Table), Interestingly, the gene for arsenical efflux pump membrane protein ArsB (BBC27_RS09700) is located separately from the *arsRCDA* operon, which is different from the *arsRDABC* operon in *E*.*coli* [37]. A set of recently illustrated arsenic resistance genes retrieved from functional metagenomic approaches were also identified [38], e.g. a phospholipid metabolism-associated gene and three genes coding for RNA-modification enzymes (S3 Table); iii) genes for copper resistance: a gene (BBC27_RS13630) coding for a copper-translocating P-type ATPase (CopB) related to the transport of copper from the cytoplasm to the periplasmic space, and four clusters of putative *cusCBA* genes coding for the Cus systems which transfers copper directly to the extracellular space [22,39]; iv) genes associated with major facilitator family (MFS) / multidrug / resistance-nodulation-cell division (RND) transporters and efflux protein (S3 Table). These genes are mainly for removal of ions like Mg^2+^, Co^2+^, Cd^2+^ and Zn^2+^. The genes for Mg^2+^ efflux are overrepresented in genome of strain YL15. This is in accordance with the fact that the concentration of Mg^2+^ in the acid mine drainage where strain YL15 inhabits achieves as high as 249 mg·l^-^, which is much higher than that in other minesites, e.g. three copper mines in central Norway [40].

#### Adaptation to low pH

Owing to the natural proton concentration gradient across the membrane in the acid mine drainage, if uncontrolled, influx of protons may lead to drastic disturbances of the intracellular pH homeostasis. In order to grow at low pH environments, acidophilic microorganisms have to maintain a pH gradient of several pH units across the cellular membrane [41]. Acidophiles achieve this via several ways, which mainly include: (i) generate a reversed membrane potential to inhibit the influx of protons via active influx of K^+^ or other cations; (ii) develop highly impermeable cell membranes to limit the influx of protons into cells; (iii) carry protons out of cells via various transporters and (iv) employ chemicals as buffer to bind and sequester protons [42].

Genome of strain YL15 harbors genes coding for a kdp-type potassium uptake ATPase system (*kdpEFABC*, S3 Table). By this means, cells of YL15 are capable of partially deflecting the inward flow of protons [41]; Membrane lipid components are known to maintain pH homeostasis in acidophiles. Hopanoid, a type of bacterial membrane lipid structures, is regarded as a critical strategy for microbial survival in extremely acidic environments [43]. A cluster of hopanoid-synthesis genes were identified in genome of YL15, e.g. a squalene/phytoene synthase gene (BBC27_RS14705), a squalene-hopene cyclase coding gene (*SHC*, BBC27_RS14700) and a number of genes coding for hopanoid-associated proteins (*HpnAIJKNHM*, S3 Table); Besides, several genes for producing of buffer molecules, e.g. genes for arginine and glutamate decarboxylase Adi and GadB (BBC27_RS02605 and 04705), exist in the genome of strain YL15. Furthermore, the existence of sodium:proton antiporter genes (BBC27_RS09220 and 12540) confirmed that cells can export excess protons and simultaneously uptake Na^+^ to cope with an increase of the intracellular proton concentration.

Some recently illustrated acid resistance genes, such as the *ClpXP* gene (BBC27_RS08380 and 08385) coding for an ATP-dependent Clp protease and the *lexA* (BBC27_RS08345) gene for a repressor protein, were found in YL15 genome [44]. It is noted that the *clpB* gene and the RNA-modification enzyme genes, which are involved in arsenic resistance, were also proved to confer acid resistance to microbes [38]. ClpB proteins are also known to be crucial in microbial adaptation to oxidative stress, suggesting its versatility in cell survival [45,46].

#### Resistance to elevated UVR

Environments in high altitude regions are typically characterized by elevated UVR. Excessive or intense exposure to UVR is detrimental to organisms [47]. Some microorganisms survive under radiation due to defensive mechanisms provided by a variety of UV-absorbing substances, e.g. mycosporine-like amino acids (MAAs), which are the secondary metabolic products in many organisms. The precursor of MAAs, 3-dehydroquinate, is formed during the early stages of the shikimate pathway [47]. Strain YL15 is presumably to produce MAAs to combat UVR, as the genes for 3-dehydroquinate synthesis are found in the genome of strain YL15. The genes are BBC27_RS08435 and 13320 (for 3-deoxy-7-phosphoheptulonate synthase) and BBC27_RS09460 (for 3-dehydroquinate synthase).

UVR leads to the production of reactive oxygen species (ROS), therefore the ROS-scavenging metabolite and/or enzymes are supposed to function in UVR-resistance. Except for MAAs, superoxide dismutase has been known to link to survival of organisms under radiation [48]. In genome of strain YL15, one copy of gene for superoxide dismutase (BBC27_RS13900) was identified. UVR causes mutagenic and cytotoxic DNA lesions [49]. Strain YL15 has dozens of DNA repair-associated genes, many of which have been substantiated to be induced by UVR, e.g. the UVr ABC system and a variety of Rec proteins (S3 Table). In addition, some other proteins, for instance, the histone-like DNA binding protein HU and Hsp70 protein (dnaK), have also been supposed to confer resistance to radiation [48], and their coding-genes are also identified in genome of strain YL15 (S3 Table).

#### Clustered regularly interspaced short palindromic repeats (CRISPR)/CRISPR-associated (Cas) gene systems

**Fig 2:**
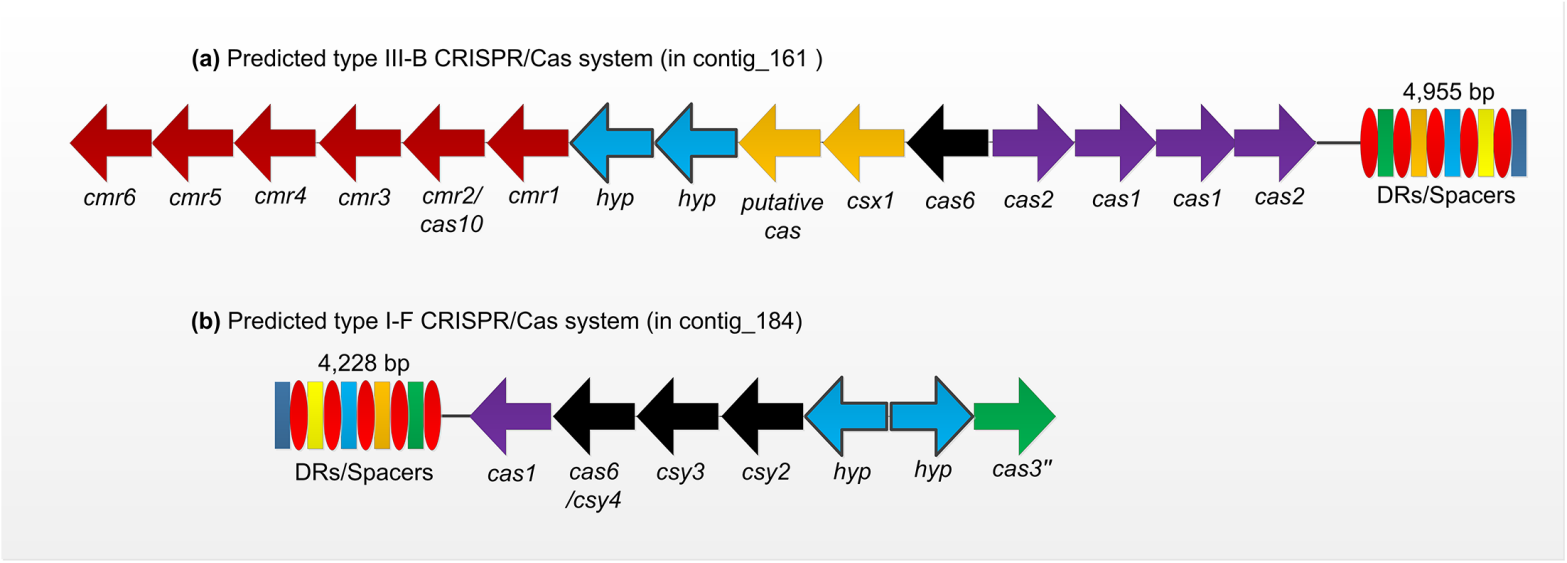
Proposed CRISPR/Cas systems in genome of YL15. The genome of YL15 harbors **(a)** a putative type III-B and **(b)** a putative type I-F CRISPR/Cas system. The type III-B system has a cluster of genes for repeat-associated mysterious proteins (RAMPs, Cmr—Cmr6, with Cmr2/Cas10 as a signature protein), a putative Cas protein and a Cas6 protein which is involved in CRISPR transcript processing. The type I-F CRISPR/Cas system has genes encoding for a Cas1 protein, three csy proteins and a Cas3” protein which has only the HD domain of Cas3 protein. Both of the systems have genes coding for two hypothetical proteins (hyp) with unknown functions.

The CRISPR/Cas (clustered regularly interspaced short palindromic repeats/CRISPR-associated genes) systems are adaptive immunity systems that are developed by prokaryotes to protect cells against foreign genetic elements such as viruses and plasmids [50]. The genome of YL15 has 2 CRISPR/Cas loci, both of which have 70 spacers (Fig 2A, 20,113 bp, Fig 2B, 13,095 bp). To the best of our knowledge, the repeats/spacers outnumber most of the known acidophilic bacteria (S4 Table). In order to identify the types of CRISPR/Cas systems, we annotated the Cas genes using Hmmscan program of HMMER package 3.1 [51] against the Pfam database 30.0 [52] and BLASTP program against the nr database. According to the classification and nomenclature of CRISPR-associated genes, the two CRISPR/Cas systems are presumed to be affiliated to type III-B targeting DNA and I-F targeting both DNA and RNA, respectively [53]. Interestingly, the type III-B system has a gene (*csx1*) for a Cas-NE0113 family protein which has been reported in type III-U system and two copies of genes for Cas1/Cas2 proteins (Fig 2 and S5 Table).

CRISPR/Cas systems are considered to function via a RNA-silencing-like mechanism, and the spacer sequences are often found to share high similarities with virus or plasmid sequences [54]. However, our BLASTN results showed that except for one spacer sequence (spacer 39, CCTATCAACGATTCGCCAATACTATCGATGTG) in the type I-F system has a similarity of 93% to a fraction of the *Sulfuricurvum kujiense* DSM 16994 plasmid pSULKU02 sequence (90% coverage), there were no other exact or full-length matches to any known phage or plasmid sequences. This may be due to that our knowledge of phage or plasmid in the acid mine drainage environments was limited and only a small fraction of their sequences have been deposited in the databases. Bacteriophages are the most abundant forms of life on the Earth, and the phage abundance is estimated to be about 5–10 times more than that of bacteria in the ocean [55]. The large number of spacers in the two CRISPR/Cas modules indicates that strain YL15 may encounter a complicated biological context. Intrusions of bacteriophage and plasmid elements are common and often lethal [56]. YL15’ s CRISPR/Cas systems help to defend against phage and plasmid invasions and thus are indispensable to its survival in the acid mine drainage environment.

### RNA-seq to reveal cold adaptation mechanisms of strain YL15

#### Overview of RNA-seq data and genes with significantly different RNA transcript counts at 28°C and 6°C

In order to gain deep insights into the cold adaptation mechanisms of strain YL15, we performed RNA-seq analysis from two biological replicates for cells grown at 28°C (S28_rep1 and S28_rep2) and at 6°C (S6_rep1 and S6_rep2). A total of 5.21 Gb clean data was created after removing reads with adapter, poly-N and low quality reads (Table 1). More than 91% of all reads were mapped to the genome of YL15 (Table 1). Overall differences in RNA transcript counts were observed between S28 and S6. A total of 372 genes with significantly different RNA transcript counts were identified, of which 199 and 173 had higher and lower RNA transcript counts at 6°C, respectively (S6 Table).

**Table 1:** Overview of Illumina RNA-seq data quality. Clean data was obtained from raw data by removing reads containing adapter, poly-N and low quality reads.

Sample name	Raw data (Gb)	Clean data (Gb)	Clean reads	Percent of reads mapped
S28_rep1	1.41	1.33	3451903	92.4
S28_rep2	1.20	1.14	2971511	92.9
S6_rep1	1.37	1.30	3380233	93.1
S6_rep2	1.53	1.44	3748436	91.1

To examine the expression of genes identified from RNA-seq analysis, quantitative real-time PCR (qRT-PCR) was performed on 25 selected genes. Glyceraldehyde-3-phosphate dehydrogenase gene (*gapdh*, BBC27_RS12850) was used as a reference since changes of the gene’s RNA transcripts were very small at 6°C and 28°C. It was shown that the correlation coefficient (*r*-value) between RNA-seq and qRT-PCR data was calculated as 0.84 (S2 Fig). This indicates the suitable quality of the RNA-seq data.

**Fig 3:**
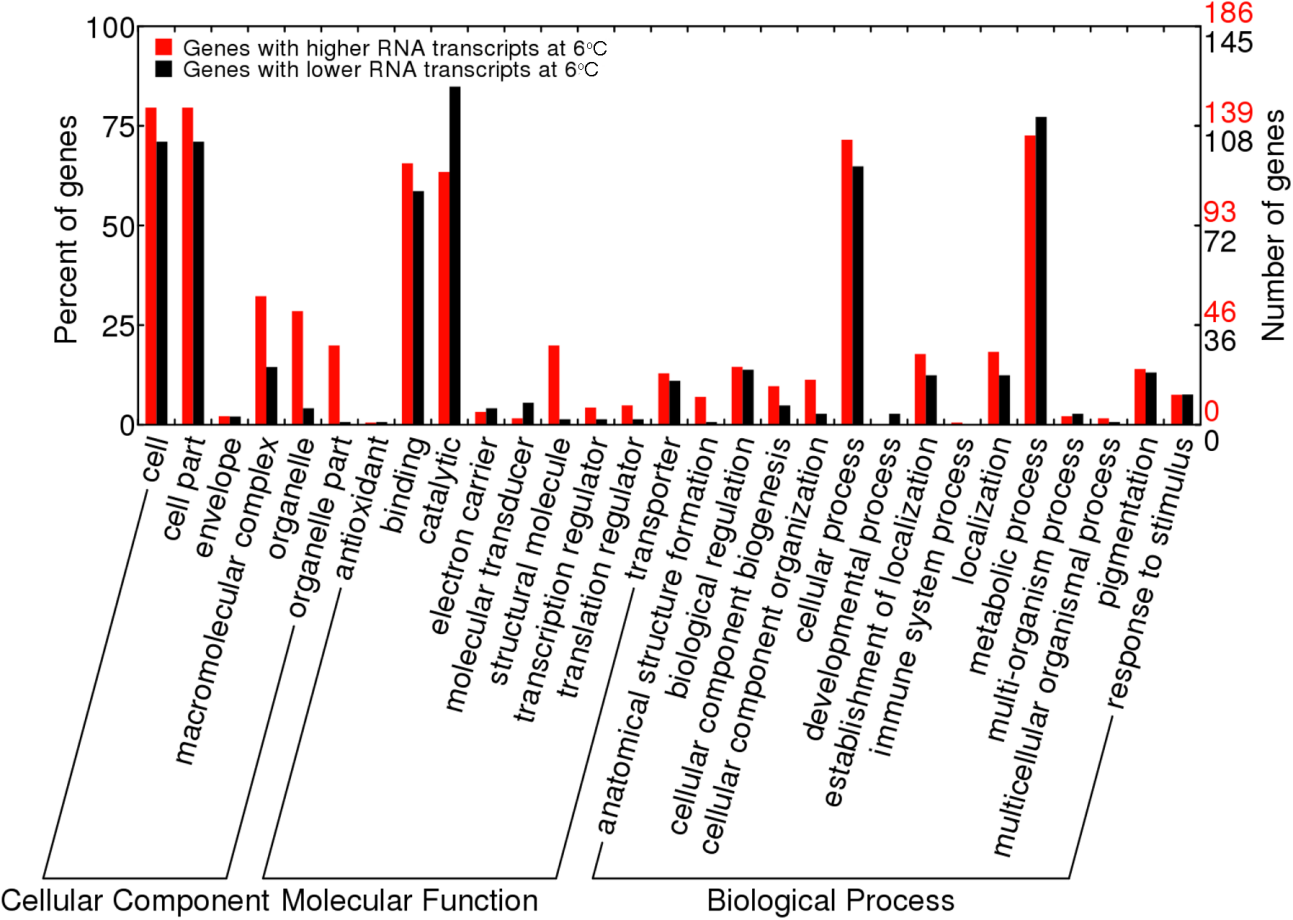
GO enrichment analysis of the differential expressed genes between S28 and S6. The numbers in red and black on the right represent the number of genes with higher and lower RNA transcripts at 6^°^C, respectively.

**Fig 4:**
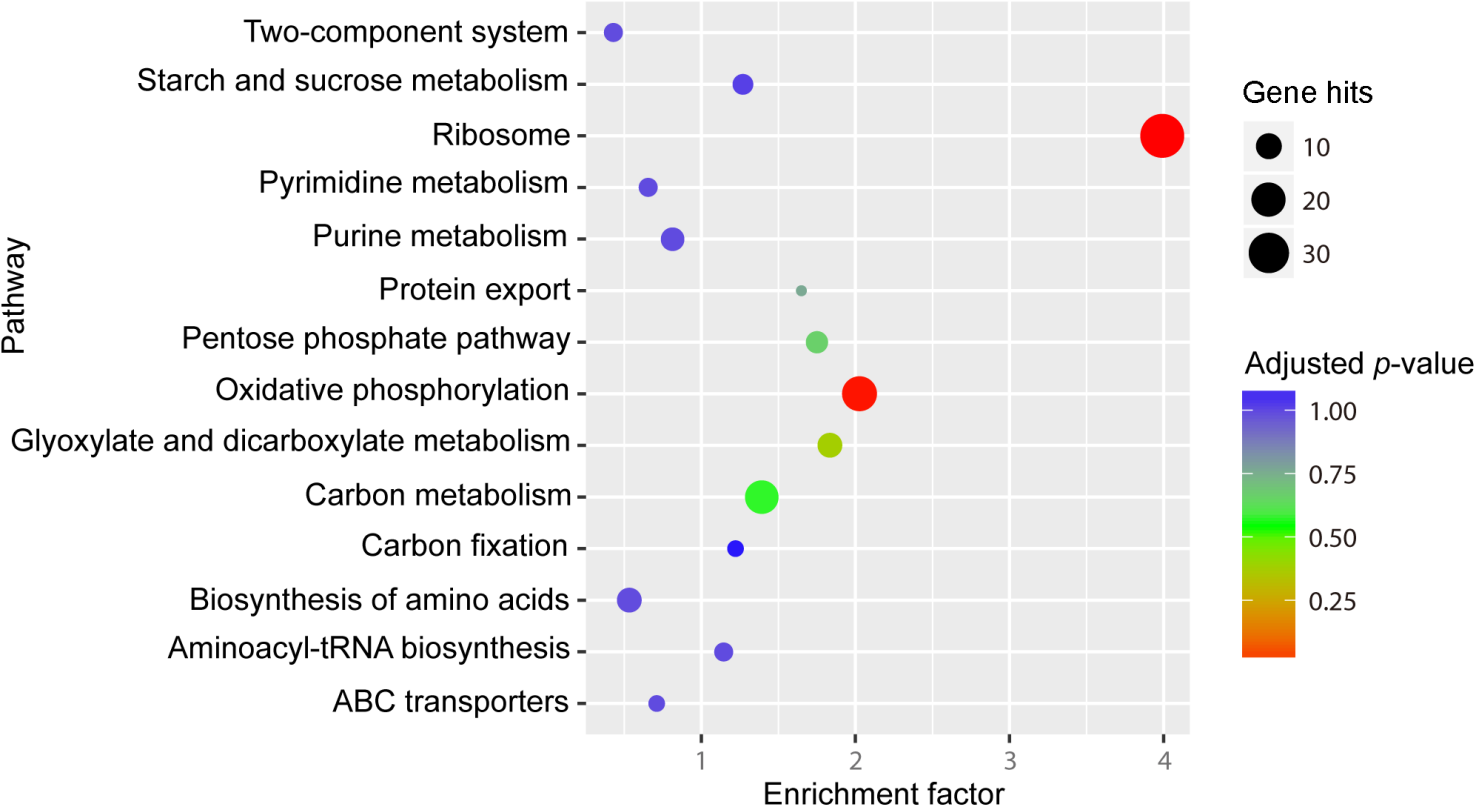
KEGG pathway enrichment analysis of differentially expressed genes between S28 and S6. Enrichment factor is calculated as followed:
$$\mathrm{Enrichment}\;\mathrm{factor}=\frac{\mathrm{Gene}\;\mathrm{hits}/{\mathrm{Gene}}_{\mathrm{pathway}}}{{\mathrm{Hits}}_{\mathrm{total}}/{\mathrm{Gene}}_{\mathrm{total}}}$$
Gene hits is the number of hits in the selected pathway; Gene_pathway_ is the number of genes in the selected pathway of KEGG background; Hits_total_ represents the number of total hits in all pathways; Gene _total_ is the number of total genes in all pathways of KEGG background.

For the purpose of acquiring the functional classification of the identified genes, gene ontology (GO) and KEGG pathway enrichment analyses were performed. The genes with significantly different RNA transcript counts were assigned to 29 functional categories by GO enrichment analysis. Among the three main GO categories, namely cellular component, molecular function and biological process, “cell” and “cell part”, ‘‘binding” and “catalytic”, “cellular process” and “metabolic process” were the most dominant subcategories, respectively. It was also found that most of the subcategories in the cellular component and biological process are up-regulated at 6°C (Fig 3). KEGG pathway enrichment analysis showed that the most dominant pathways were ribosome, oxidative phosphorylation and carbon metabolism (Fig 4).

#### Transcription

A number of genes involved in transcription showed increased number of RNA transcript counts in cold condition compared to those in mesophilic condition. At 6°C, most of the genes for RNA polymerase complex core enzyme subunits (alpha, beta and beta’) had higher RNA transcripts. In particular, the beta subunit coding gene had 5.44- fold more RNA transcript counts. The up-regulation of RNA polymerase has been observed in some other microorganisms, e.g. a marine bacterium *Sphingopyxis alaskensis* [57] and a methanogenic archaeum *Methanolobus psychrophilus* R15 [58] and also in *A*. *ferrivorans* strain SS3 [9]. Besides, the gene coding for RNA polymerase sigma factor RpoD was also induced by cold. Several transcription factor genes showed cold-enhanced RNA transcript counts, including those coding for a transcriptional initiation protein Tat, a transcription elongation factor GreA, a transcription termination factor Rho and transcription-repair coupling factor. Moreover, the YL15 genome has three genes coding for transcription antitermination protein, and two of them (*nusA a*nd *nusG*) had higher RNA transcript levels at 6°C. The elevated RNA transcript levels of cellular components of the transcriptional machinery at low temperature, together with the induction of genes involved in the transcriptional processes by cold, demonstrated that transcriptional regulation is central to cold adaptation in strain YL15.

#### Translation and post-translational processing

A large quantities of genes that are involved in translation and post-translational processing had greater number of RNA transcript levels at 6°C. Genes for 37 ribosome proteins, including genes for 24 large subunit and 13 small subunit proteins, and a ribosome maturation factor showed increased RNA transcript counts at 6°C. In addition, the translation initiation factors (IFs) have been found to be related to translation of cold-induced genes [59]. The genes for the translation initiation factors, IF-1 and IF-3, had 3.59 and 5.06-fold more RNA transcript counts at 6°C, respectively. Besides, a gene coding for the translation elongation factor G, which catalyzes the translocation of the tRNA and mRNA down the ribosome at the end of each round of polypeptide elongation, also had elevated RNA transcript counts at 6°C.

The rise of RNA transcript counts for genes involved in translation may reflect a requirement for more proteins to cope with the cold-stress conditions. Nevertheless, it has been speculated that some ribosomal proteins may have other functions apart from protein synthesis, such as acting as a temperature sensor to cold stress [60]. Moreover, ribosomal proteins have also been found to be bacterial surface and secreted proteins, thus it was inferred that some ribosomal proteins may be secreted to the surface of the cells or out of cells as a defensive mechanism in response to external environmental changes [61].

RNA transcript counts for some genes encoding for factors involved in posttranslational processing were affected by changes of temperature. A dozen of chaperone-encoding genes were found in the YL15 genome, including the group I chaperonin complex (GroEL/ES) coding genes and genes for molecular chaperone HtpG, dnaK and dnaJ. RNA transcript count for the HtpG-coding genes was elevated at 6°C. Another chaperone gene, coding for a peptidylprolyl isomerase (PPIase) was also induced by temperature downshift. It has been found that PPIase enhances protein folding by catalyzing the rate-limiting cis/trans isomerization of peptidyl-prolyl bonds in polypeptides, and some PPIases are also capable of refolding unfolded proteins [62].

#### Transmembrane transport

Numerous ABC transporter-associated genes were identified in the genome of strain YL15, 4 of which showed increased number of RNA transcript counts in the cold. Notably, genes (*pstSCA*) for the high-affinity ABC-type phosphate uptake system in the genome had greater number of RNA transcript counts at 6°C. The increases in the RNA transcript counts for this high-affinity system suggest an urge demand for maintaining a sufficient supply of phosphate for use in central metabolic actions at low-temperatures (e.g. DNA replication and protein synthesis). This may also reflect a decline in transport efficiency and that the increased transcript levels of these genes might compensate for reduced enzyme activity at low temperatures.

Proteins can be exported out of the cytoplasmic membrane via different pathways, e.g., the Sec protein-translocation pathway. The Sec translocases offer a major pathway of protein translocation from the cytoplasm across the cell membrane in bacteria [63]. Genome of YL15 has genes for Sec pathway (*secYABDEFGyajC*), and four of them (*secYEFyajC*) showed greater RNA transcript counts at 6°C. It has been found that a large number of surface proteins including secreted proteins were more abundant at 4°C than at 23°C in a psychrophilic archaeum *Methanococcoides burtonii* [64]. The roles that the secreted proteins play remains obscure, they may facilitate intercellular interactions that promote nutrient exchange under unfavorable environments, improve the stability of the cell membrane, and/or act as a part of extracellular polymeric substances (EPS) to cope with the low temperatures.

#### Energy metabolism

Previous studies have shown that genes involved in energy metabolism are induced by cold [65]. In *A*. *ferrivorans* strain SS3, eight genes involved in inorganic sulfur compounds oxidation and 20 electron transport genes had higher RNA transcript counts at 8^°^C [9]. In contrast, in strain YL15, most of the genes for F_0_F_1_ ATP synthase had higher RNA transcript levels at low temperature. It was found that the genes for F_0_F_1_ ATP synthase subunit A, B, C, alpha, gamma and delta had increased number of RNA transcript counts at 6°C. In *A*. *ferrivorans*, the synthesis of ATP via F_0_F_1_ ATP synthase is coupled with the ferrous iron pathway. The downhill pathway of ferrous iron oxidation can consume protons entering the cells via the ATP synthase complex and drive ATP synthesis [6,66]. It was observed that the gene coding for the key enzyme short-chain dehydrogenase in the iron-oxidation pathway, showed a rise in RNA transcript counts at low temperature. These results indicate that strain YL15 has higher energy demands at low temperatures, to fuel the increased production of specific proteins and enhanced metabolic processes required to deal with cold conditions.

#### Chemotaxis and motility

Bacterial chemotaxis is expected to enable cells to move towards favorable environments and evade unfavorable conditions. Three genes involving in chemotaxis, namely chemotaxis phosphatase gene *CheZ*, and a gene for a methyl-accepting chemotaxis protein, had more RNA transcript counts at 6^°^C. The induction of chemotaxis genes upon long-term adaptation to low temperature has been found in the human pathogen *Yersinia enterocolitica* [67]. It can be inferred that cell motility may also be cold-enhanced since bacterial chemotaxis relies on cell motility. The genome of YL15 has a set of genes for assembly and function of the microbial motility organelle–flagellum. The bacterial proteins MotA and MotB are required for the rotation of the flagellar motor [68,69]. The genome of YL15 has five and three copies of genes coding for MotA and MotB proteins, respectively. Two of the MotA gene (BBC27_RS01105, 02275) had higher RNA transcript levels at low temperature. Particularly, one of the MotA gene (BBC27_RS01105) had 7.25-fold rise in RNA transcript counts at 6°C. In addition, except for one gene (*FlhB*, BBC27_RS10385), the other genes for flagellum assembly had no significantly difference in RNA transcript counts at 6°C and 28°C. These results indicated that the Mot genes may play a critical role and flagella in cells of YL15 may function with a unique mechanism in response to cold.

#### Other mechanisms

Cold shock proteins (Csps) function in bacterial survival in various adverse conditions including rapid temperature downshifts [70,71]. Csps are thought to function by serving as RNA chaperons that may prevent the formation of mRNA secondary structures at low temperatures and thus facilitate translation [72]. We found that one Csp gene (BBC27_RS12050) had an elevated RNA transcript level at 6°C. The strain YL15 has been acclimated for months before our experiment, therefore, the Csp gene is also a cold acclimation protein (Cap) gene, which was induced during balanced growth at low temperatures [73]. Moreover, the gene had high RNA transcript counts at both 6°C and 28°C, suggesting that the gene was responsible for not only cold adaptation but also survival of strain YL15 at mesophilic temperature.

It is noted that a gene coding for a bacterioferritin (also known as DNA binding proteins from starved cells, Dps) had a 10-fold increase in RNA transcript counts at 6°C. Dps is thought to protect DNA against oxidative stress mediated by H_2_O_2_. H_2_O_2_ is involved in Fenton reaction, a Fe^2+^-facilitated chemical reaction to generate hydroxyl radical, which is a type of reactive oxygen species (ROS) that are detrimental to organisms [74,75]. Elevated concentration of H_2_O_2_ can be anticipated due to increased solubility of oxygen at low temperatures [11,74]. This indicates that Dps in strain YL15 may aid cells in protecting DNA from oxidative damages.

**Fig 5:**
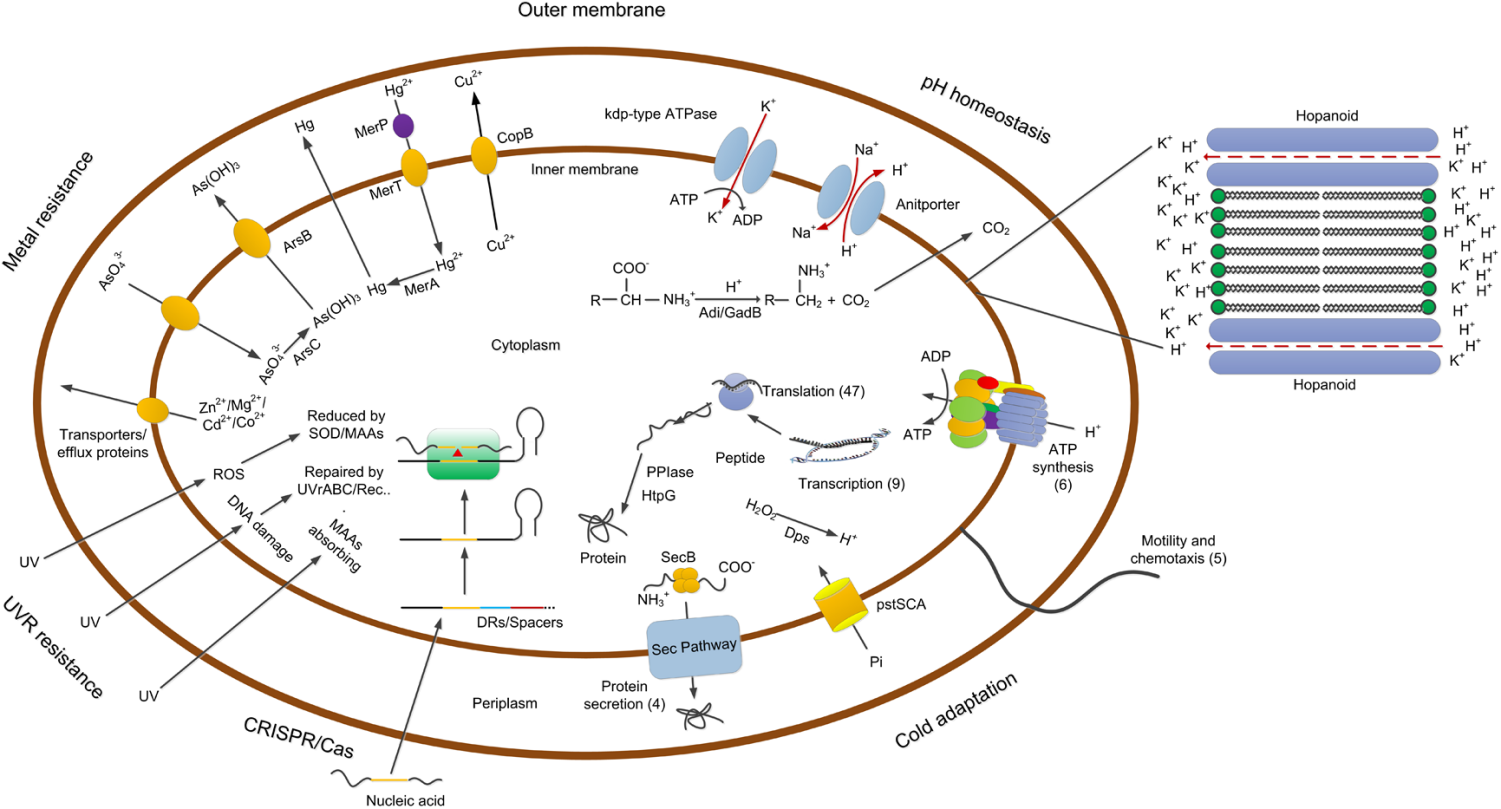
Proposed model for processes involved in adaptation of YL15 to the alpine acid mine drainage. The structure of hopanoid was shown in detail on the right. The numbers in parentheses represent the number of genes with higher RNA transcripts at 6°C versus 28°C.

In summary, this report represents the first comprehensive study of the microbial adaptation mechanisms to the alpine acid mine drainage environments at a strain-level. Microorganisms in the acid mine drainage environments are faced with severe survival threats either abiotic or biotic. The main survival pressures include resistance to metal ions, maintaining a near-neutral intracellular pH and precluding invasion of extraneous nucleic acid substances. However, for strains like YL15 that inhabit acid mine drainage in alpine regions, additional vital challenges are adaptation to low temperatures and resistance to UVR. Genomic analysis illustrated the metal-tolerance, pH homeostasis and UVR-resistance mechanisms and the acquired immune system—CRISPR/Cas modules of strain YL15. For the first time, we characterized the potential UVR-resistance mechanisms and the CRISPR/Cas systems with a large number of repeats/spacers in an acidophile. Transcriptomic assays revealed the cold adaptation mechanisms of the strain. The results also demonstrated that although our results are consistent with those of Christel et al. [9] in some aspects, e.g. changes in transcript counts for genes for RNA polymerase complex and translation regulation, considerable differences lie in variation of transcript levels for genes involved in ribosomal proteins, energy metabolism, chemotaxis and motility and biofilm formation (S7 Table). These differences may be the results of differences in the energy sources, the temperatures and / or the strains adopted. On the basis of our analyses, a schematic model for selected gene products and / or processes involved in adaptation of strain YL15 to the alpine acid mine drainage environment was proposed (Fig 5). This study adds to our knowledge on the mechanisms by psychrophilic acidophiles to deal with the mine-affected water bodies and also sheds light on the adaptation mechanisms of other microorganisms to the extreme environments.

## Supporting information

S1 Fig Growth curves of *A*. *ferrivorans* YL15 at 6°C and 28°C. Filtration sterilized ferrous sulfate was used as an energy source.
(TIF)

S2 Fig Correlation analysis of RNA-seq and qRT-PCR. (TIF)

S1 Table Physicochemical properties of the acid mine drainage sample for strain YL15. (DOCX)

S2 Table Selected genes and primers for quantitative real-time PCR. (DOCX)

S3 Table Genes predicted to involved in metal resistance, pH homeostasis and UVR-resistance. The symbol ‘/’means the genes has no specific functions.
(DOCX)

S4 Table Comparison of CRISPRs loci among acidophilic bacteria. CRISPRs loci of YL15 were annotated using CRISPRFinder while those of other strains were retrieved from CRISPRs database.
(DOCX)

S5 Table Identification of CRISPR-associated (Cas) proteins in strain YL15. (DOCX)

S6 Table Functional genes with differential transcritpts based on analysis by TopHat and Cufflinks packages. There were 199 and 173 genes with significantly higher and lower RNA transcripts out of the total 2,798 protein-coding genes in genome of strain YL15. Classification of protein functions was based on KEGG annotation.
(DOCX)

S7 Table Comparison of number of genes in main categories with higher transcripts levels at low temperatures between results from Christel et al. and this study. The bold and regular letters represent categories and sub-categories, respectively. Sub-categories were based on classification of protein functions as listed in S6 Table.
(DOCX)
