# Supplementary figures and images for: Genomic and transcriptomic analyses reveal adaptation mechanisms of an Acidithiobacillus ferrivorans strain YL15 to alpine acid mine drainage
Source: PLoS One. 2017 May 19;12(5):e0178008. doi: 10.1371/journal.pone.0178008 (PMC5438186; doi:10.1371/journal.pone.0178008)

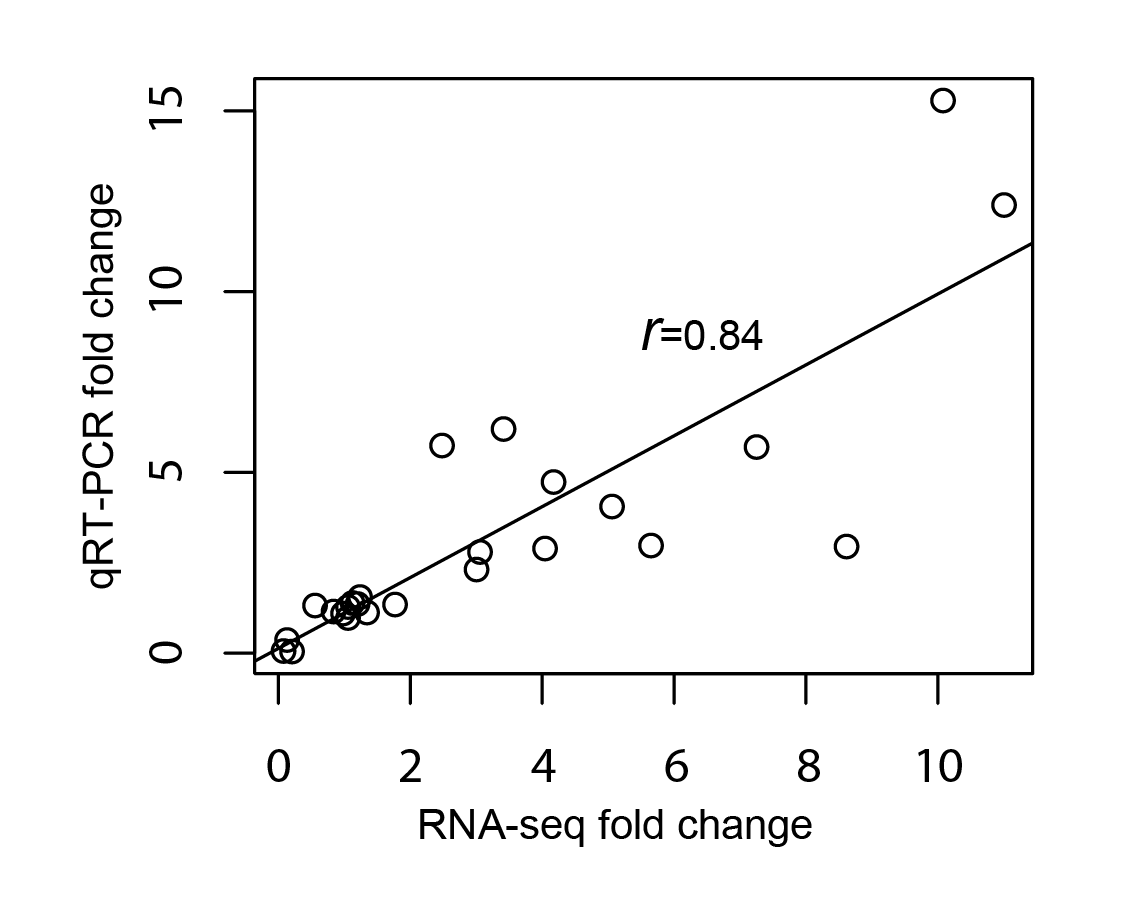

Supplement: S2 Fig — (TIF) [file pone.0178008.s002.tif]
