# Supplementary material for: Genomic and transcriptomic analyses reveal adaptation mechanisms of an Acidithiobacillus ferrivorans strain YL15 to alpine acid mine drainage
Source: PLoS One. 2017 May 19;12(5):e0178008. doi: 10.1371/journal.pone.0178008 (PMC5438186; doi:10.1371/journal.pone.0178008)
Supplement: S1 Table — (DOCX) [file pone.0178008.s003.docx]

**S1 Table.** **Physicochemical properties of the acid mine drainage sample for strain YL15.**

| pH | Temperature (^o^C)* | Concentration (mg^.^l^-^) | | | | | | | | | |  |
| --- | --- | --- | --- | --- | --- | --- | --- | --- | --- | --- | --- | --- |
|  |  | Total iron | Al | Cu | Cr | Co | Pb | Zn | As | Mg | Ca | Mn |
| 2.94 | 10 | 273 | 263 | 1014 | 0.17 | 5.93 | 3.39 | 75.53 | 1.14 | 249 | 202 | 112.4 |

* Temperature measured while sampling.
