# Supplementary material for: Genomic and transcriptomic analyses reveal adaptation mechanisms of an Acidithiobacillus ferrivorans strain YL15 to alpine acid mine drainage
Source: PLoS One. 2017 May 19;12(5):e0178008. doi: 10.1371/journal.pone.0178008 (PMC5438186; doi:10.1371/journal.pone.0178008)
Supplement: S2 Table — (DOCX) [file pone.0178008.s004.docx]

**S2 Table**. **Selected genes and primers for quantitative real-time PCR.**

| Gene | Protein | Primers | Primer sequences (5’-3’) | Amplicon length (bp) |
| --- | --- | --- | --- | --- |
| BBC27_RS01105 | Flagellar motor protein MotA | Mot-F | TTCCCATCACGGACAGGTC | 149 |
|  |  | Mot-R | TAATCGTGCCGAACAAACC |  |
| BBC27_RS01890 | Transporter | Trans-F | TCTTCACTGGCTTCAATACGCATTAT | 104 |
|  |  | Trans-R | CGTCGCTTCGGTGCTGTTG |  |
| BBC27_RS03710 | Molecular chaperone HtpG | Htp-F | AGCGGCGATCAGACCAAAG | 141 |
|  |  | Htp-R | CGACTCCCAGCGAATACCAT |  |
| BBC27_RS06525 | Lipid carrier-like protein | Lipc-F | GCGGATTTCCATCAGATAGCG | 175 |
|  |  | Lipc-R | CTTCGAGGCGTAGTGCGAGT |  |
| BBC27_RS09040 | Bacterioferritin | Dps-F | CTGTACGAAGAAGCCGCCAACT | 111 |
|  |  | Dps-R | GCCATCCAGATCACGCAAG |  |
| BBC27_RS12050 | Cold-shock protein | Csp-F | CAACTGGCACAGTAAAGTGGTTC | 105 |
|  |  | Csp-R | TGCCCTGGATAGCAGAGTGAT |  |
| BBC27_RS14205 | Hypothetical protein | RS14205-F | CTGCTCTGCCGCCTCATACTCT | 83 |
|  |  | RS14205-R | CCGTCCTGAATCTGGCTTTGG |  |
| BBC27_RS14280 | Rusticyanin | Rus-F | TGGACTTCAAAGCTCGGGAATG | 204 |
|  |  | Rus-R | GGCGGTCACTGTGGGTATGG |  |
| BBC27_RS02885 | Glycine cleavage system protein H | Glycle-F | ATGACCTCCTATGCCTGTGCG | 139 |
|  |  | Glycle -R | TGACGGGACTCTTCAACGG |  |
| BBC27_RS14305 | Hypothetical protein | RS14305-F | TAATGACTTGCCCAAATGGAC | 109 |
|  |  | RS14305-R | GGTGCGACTACGGTGCTAAT |  |
| BBC27_RS07200 | Iron-sulfur cluster scaffold-like protein | Irsusca-F | TGCTTGTTGCGGTAATCCTCG | 181 |
|  |  | Irsusca -R | CGGCTATTGCGTCCAGTTCG |  |
| BBC27_RS10970 | Hypothetical protein | RS10970-F | CGGTGAGTAGCCTCGCTTTACTG | 138 |
|  |  | RS10970-R | GCATCCACAGAATAATCCCTAGAGC |  |
| BBC27_RS14810 | Heterodisulfide reductase  subunit B, partial | HdrB-F | GACAACAGCACGCAGCAGG | 214 |
|  |  | HdrB-R | GATCGTCGGTGATACGGTGG |  |
| BBC27_RS04065 | Glucan biosynthesis protein D | Glucan-F | GTCAAGATTGCGGTTATTGGTAGG | 134 |
|  |  | Glucan-R | GGCGATTCACCAGTTATGAGG |  |
| BBC27_RS11770 | Isoprenylcysteine carboxyl methyltransferase | Isocam-F | GAAGGCATTCGTCAGGATGTG | 106 |
|  |  | Isocam-R | GCTGTCGTTTGGTTGCTTG |  |
| BBC27_RS02015 | 50S ribosomal protein L10 | L10-F | GCAGTGGTGGCAGAGTATCG | 228 |
|  |  | L10-R | CTTTGCGTGGTCGGTGAAG |  |
| BBC27_RS01390 | Phosphate ABC transporter substrate-binding protein PstS | Pst-F | CCTGTGCCCTGGGTGGTAAT | 141 |
|  |  | Pst-R | GTATGCCCTGCCTGCTTCG |  |
| BBC27_RS03165 | Translation initiation factor IF-1 | Transif-F | CGATAAATGATGCGACCCT | 205 |
|  |  | Transif-R | CGAAAGAAGATACCCTGGAAAT |  |
| BBC27_RS03215 | 50S ribosomal protein L24 | L24-F | CACTTTCTGCCCGTCCTCA | 150 |
|  |  | L24-R | CGGCACGTTCGTCCTGAT |  |
| BBC27_RS14785 | Elongation factor Tu | Tu-F | TACGTCGGTGGTACGGAAGT | 227 |
|  |  | Tu-R | GCAAGATTCTGGATCAGGGTC |  |
| BBC27_RS03245 | 50S ribosomal protein L22 | L22-F | GCGACGACAACGGTGATAT | 238 |
|  |  | L22-R | TGGGTAAGGCACTGGAGAT |  |
| BBC27_RS03145 | DNA-directed RNA polymerase subunit alpha | Drpm-F | AATTGTCGAGCCGCATCC | 116 |
|  |  | Drpm-R | TCAGAAGTCGGAGCAGGAG |  |
| BBC27_RS11495 | Ribulose-bisphosphate carboxylase large subunit | Rubpl-F | GCCCAAACTCGGTCTATCTG | 240 |
|  |  | Rubpl-R | TCTGCCCGTTCATACATTTC |  |
| BBC27_RS10915 | Hypothetical protein | RS10915-F | AGTGACCTTGGCGGTGTT | 238 |
|  |  | RS10915-R | CCATCTCGTTATCGCTTTACTCAT |  |
| BBC27_RS04775 | Triose-phosphate isomerase | Tripi-F | CAGGTCACCCAGAAAGTTAAGGT | 147 |
|  |  | Tripi-R | GAGATTCAACAGCGGCAAG |  |
| BBC27_RS12850 | Glyceraldehyde-3-phosphate dehydrogenase | Gapdh-F | CCCTCGTTGACCTCACCTGTATC | 161 |
|  |  | Gapdh-R | GGTGGAGGAGTGCGGATTATGG |  |
