# Supplementary material for: Genomic and transcriptomic analyses reveal adaptation mechanisms of an Acidithiobacillus ferrivorans strain YL15 to alpine acid mine drainage
Source: PLoS One. 2017 May 19;12(5):e0178008. doi: 10.1371/journal.pone.0178008 (PMC5438186; doi:10.1371/journal.pone.0178008)
Supplement: S3 Table — The symbol ‘/’means the genes has no specific functions. (DOCX) [file pone.0178008.s005.docx]

**S3 Table.** **Genes predicted to involved in metal resistance, pH homeostasis and UVR-resistance.** The symbol ‘/’means the genes has no specific functions.

| Gene | Protein | Function |
| --- | --- | --- |
| **Metal resistance** |  |  |
| BBC27_RS11670 | Mercury transporter MerT | Mercury resistance |
| BBC27_RS11675 | Periplasmic mercuric ion-binding protein MerP | Mercury resistance |
| BBC27_RS11680 | Mercury(II) reductase | Mercury resistance |
| BBC27_RS11685 | MerR family transcriptional regulator | Mercury resistance |
| BBC27_RS04630 | MerR family transcriptional regulator | Mercury resistance |
| BBC27_RS04905 | MerR family transcriptional regulator | Mercury resistance |
| BBC27_RS11570 | Arsenical resistance operon repressor | Arsenic resistance |
| BBC27_RS11575 | ArsC family transcriptional regulator | Arsenic resistance |
| BBC27_RS11580 | Arsenical resistance operon trans-acting repressor ArsD | Arsenic resistance |
| BBC27_RS11585 | Arsenical pump-driving ATPase, partial | Arsenic resistance |
| BBC27_RS06310 | Arsenate reductase | Arsenic resistance |
| BBC27_RS09700 | Arsenical efflux pump membrane protein ArsB | Arsenic resistance |
| BBC27_RS08660 | ATP-dependent chaperone ClpB | Arsenic resistance |
| BBC27_RS09575 | tRNA guanosine(34) transglycosylase Tgt | Arsenic resistance |
| BBC27_RS09580 | tRNA preQ1(34) S-adenosylmethionine ribosyltransferase-isomerase QueA | Arsenic resistance |
| BBC27_RS09645 | Aspartate-semialdehyde dehydrogenase | Arsenic resistance |
| BBC27_RS03080 | Phosphoesterase | Arsenic resistance |
| BBC27_RS08115 | Formamidopyrimidine-DNA glycosylase | Arsenic resistance |
| BBC27_RS08120 | Sulfate permease | Arsenic resistance |
| BBC27_RS09630 | N-(5\'-phosphoribosyl)anthranilate isomerase | Arsenic resistance |
| BBC27_RS09635 | tRNA pseudouridine(38-40) synthase TruA | Arsenic resistance |
| BBC27_RS13630 | Copper-translocating P-type ATPase | Copper resistance |
| BBC27_RS15015 | Transporter/putative cusA | Copper resistance |
| BBC27_RS15020 | Efflux transporter periplasmic adaptor subunit/putative cusB | Copper resistance |
| BBC27_RS15025 | Transporter/putative cusC | Copper resistance |
| BBC27_RS01880 | Acriflavine resistance protein B/putative cusA | Copper resistance |
| BBC27_RS01885 | Efflux transporter periplasmic adaptor subunit/putative cusB | Copper resistance |
| BBC27_RS01890 | Transporter/putative cusC | Copper resistance |
| BBC27_RS02775 | Multidrug efflux protein/putative cusA | Copper resistance |
| BBC27_RS02780 | Efflux transporter periplasmic adaptor subunit/putative cusB | Copper resistance |
| BBC27_RS02785 | RND transporter/putative cusC | Copper resistance |
| BBC27_RS08295 | RND transporter/putative cusC | Copper resistance |
| BBC27_RS08300 | Efflux transporter periplasmic adaptor subunit/putative cusB | Copper resistance |
| BBC27_RS08305 | Multidrug efflux protein/putative cusA | Copper resistance |
| BBC27_RS06140 | Multidrug MFS transporter | Metal efflux |
| BBC27_RS08545 | Multidrug transporter | Metal efflux |
| BBC27_RS01500 | Magnesium/cobalt efflux protein | Metal efflux |
| BBC27_RS06645 | Cobalt transporter | Metal efflux |
| BBC27_RS09585 | Cobalt transporter | Metal efflux |
| BBC27_RS09380 | Cation transporter | Metal efflux |
| BBC27_RS11250 | DNA-binding response regulator | Metal efflux |
| BBC27_RS11255 | Two-component sensor histidine kinase | Metal efflux |
| BBC27_RS11260 | Transporter | Metal efflux |
| BBC27_RS11265 | Efflux transporter periplasmic adaptor subunit | Metal efflux |
| BBC27_RS00150 | Heavy metal-responsive transcriptional regulator | Metal efflux |
| BBC27_RS02785 | RND transporter | Metal efflux |
| BBC27_RS05655 | Co^2+^/Mg^2+^ efflux protein ApaG | Metal efflux |
| BBC27_RS06660 | MFS transporter | Metal efflux |
| BBC27_RS07645 | Multidrug ABC transporter ATP-binding protein | Metal efflux |
| BBC27_RS09585 | Cobalt transporter | Metal efflux |
| BBC27_RS10790 | Multidrug MFS transporter | Metal efflux |
| BBC27_RS10795 | RND transporter | Metal efflux |
| BBC27_RS10800 | Multidrug transporter | Metal efflux |
| BBC27_RS14370 | Multidrug ABC transporter ATP-binding protein | Metal efflux |
| BBC27_RS14905 | Efflux transporter periplasmic adaptor subunit, partial | Metal efflux |
| BBC27_RS11775 | Magnesium transporter, partial | Metal efflux |
| BBC27_RS11785 | Magnesium transporter | Metal efflux |
| BBC27_RS11790 | Magnesium transporter | Metal efflux |
| BBC27_RS12820 | Magnesium transporter | Metal efflux |
| BBC27_RS12020 | Cation transporter | Metal efflux |
| BBC27_RS13620 | Heavy metal transport/detoxification protein | Metal efflux |
| **pH homeostasis** |  |  |
| BBC27_RS02160 | DNA-binding response regulator/ kdp operon transcriptional regulatory protein kdpE | kdp-type potassium uptake ATPase |
| BBC27_RS02170 | K^+^-transporting ATPase subunit F | kdp-type potassium uptake ATPase |
| BBC27_RS02175 | Potassium-transporting ATPase subunit KdpA | kdp-type potassium uptake ATPase |
| BBC27_RS02180 | K^+^-transporting ATPase subunit B | kdp-type potassium uptake ATPase |
| BBC27_RS02185 | K^+^-transporting ATPase subunit C | kdp-type potassium uptake ATPase |
| BBC27_RS02605 | Arginine decarboxylase | Producing buffer molecules |
| BBC27_RS04575 | Aspartate 1-decarboxylase | Producing buffer molecules |
| BBC27_RS04705 | Glutamate decarboxylase | Producing buffer molecules |
| BBC27_RS09390 | Phosphatidylserine decarboxylase | Producing buffer molecules |
| BBC27_RS12310 | Spermidine synthase | Producing buffer molecules |
| BBC27_RS08345 | Repressor LexA | / |
| BBC27_RS08380 | ATP-dependent Clp protease ATP-binding subunit ClpX | / |
| BBC27_RS08385 | ATP-dependent Clp protease proteolytic subunit | / |
| BBC27_RS08660 | ATP-dependent chaperone ClpB | / |
| BBC27_RS09220 | Na^+^/H^+^ antiporter NhaA | Antiporter |
| BBC27_RS12540 | Sodium:proton antiporter | Antiporter |
| BBC27_RS06675 | Hypothetical protein/ Hopanoid biosynthesis associated membrane protein HpnM | Hopanoid biosynthesis |
| BBC27_RS14655 | Membrane protein/Hopanoid biosynthesis associated membrane protein HpnM | Hopanoid biosynthesis |
| BBC27_RS14660 | Hopanoid biosynthesis associated radical SAM protein HpnH | Hopanoid biosynthesis |
| BBC27_RS14670 | Hypothetical protein/Hopanoid biosynthesis associated RND transporter like protein HpnN | Hopanoid biosynthesis |
| BBC27_RS14675 | Hypothetical protein/Hopanoid biosynthesis associated protein HpnK | Hopanoid biosynthesis |
| BBC27_RS14680 | Hopanoid biosynthesis associated radical SAM protein HpnJ | Hopanoid biosynthesis |
| BBC27_RS14685 | Glycosyl transferase/Hopanoid biosynthesis associated glycosyl transferase protein HpnI | Hopanoid biosynthesis |
| BBC27_RS14695 | Nucleosidase/hopanoid-associated phosphorylase | Hopanoid biosynthesis |
| BBC27_RS14700 | Squalene-hopene cyclase | Hopanoid biosynthesis |
| BBC27_RS14705 | Squalene/phytoene synthase | Hopanoid biosynthesis |
| **UVR-resistance** |  |  |
| BBC27_RS08435 | 3-deoxy-7-phosphoheptulonate synthase | MAAs precursor synthesis |
| BBC27_RS13320 | 3-deoxy-7-phosphoheptulonate synthase | MAAs precursor synthesis |
| BBC27_RS09460 | 3-dehydroquinate synthase | MAAs precursor synthesis |
| BBC27_RS13900 | Superoxide dismutase | ROS-scavenger |
| BBC27_RS03885 | Excinuclease ABC subunit A, partial | DNA repair |
| BBC27_RS07555 | Excinuclease ABC subunit B | DNA repair |
| BBC27_RS07595 | Excinuclease ABC subunit C | DNA repair |
| BBC27_RS05035 | DNA repair protein RecO | DNA repair |
| BBC27_RS06575 | DNA recombination protein RecF | DNA repair |
| BBC27_RS11020 | DNA recombination/repair protein RecA | DNA repair |
| BBC27_RS11340 | ATP-dependent DNA helicase RecG, partial | DNA repair |
| BBC27_RS06085 | DNA mismatch repair protein MutS | DNA repair |
| BBC27_RS14030 | DNA mismatch repair protein MutL | DNA repair |
| BBC27_RS05990 | DNA repair protein RadA | DNA repair |
| BBC27_RS13065 | Holliday junction DNA helicase RuvB | DNA repair |
| BBC27_RS13350 | Molecular chaperone DnaK/Hsp70 | DNA repair |
| BBC27_RS04350 | Integration host factor subunit alpha/histone-like DNAbinding protein HU | DNA repair |
