# Supplementary material for: Genomic and transcriptomic analyses reveal adaptation mechanisms of an Acidithiobacillus ferrivorans strain YL15 to alpine acid mine drainage
Source: PLoS One. 2017 May 19;12(5):e0178008. doi: 10.1371/journal.pone.0178008 (PMC5438186; doi:10.1371/journal.pone.0178008)
Supplement: S4 Table — CRISPRs loci of YL15 were annotated using CRISPRFinder while those of other strains were retrieved from CRISPRs database. (DOCX) [file pone.0178008.s006.docx]

**S4 Table.** **Comparison of CRISPRs loci among acidophilic bacteria.** CRISPRs loci of YL15 were annotated using CRISPRFinder while those of other strains were retrieved from CRISPRs database.

| Strain | No. of CRISPRs locus | No. of spacers |
| --- | --- | --- |
| YL15 | 2 | 70, 70 |
| *Acidithiobacillus ferrivorans* CF27 | 1 | 17 |
| *Acidithiobacillus ferrooxidans* ATCC 23270 | 4 | 1, 4, 7, 3 |
| *Acidithiobacillus ferrooxidans* BY0502 | 1 | 5 |
| *Acidiphilium cryptum* JF-5 | 2 | 39, 3 |
| *Acidobacterium capsulatum* ATCC 51196 | 1 | 23 |
| *Acidithiobacillus caldus* SM-1 | 3 | 2, 14, 11 |
| *Acidimicrobium ferrooxidans* DSM 10331 | 2 | 27, 41 |
| *Sulfobacillus acidophilus* TPY | 2 | 7, 7 |
| *Sulfobacillus acidophilus* DSM 10332 | 2 | 42, 26 |
