# Supplementary material for: Genomic and transcriptomic analyses reveal adaptation mechanisms of an Acidithiobacillus ferrivorans strain YL15 to alpine acid mine drainage
Source: PLoS One. 2017 May 19;12(5):e0178008. doi: 10.1371/journal.pone.0178008 (PMC5438186; doi:10.1371/journal.pone.0178008)
Supplement: S5 Table — (DOCX) [file pone.0178008.s007.docx]

**S5 Table.** **Identification of CRISPR-associated (Cas) proteins.**

| Locus tag | Product Length | Similar protein (organism) | Identity  (%, blastp) |
| --- | --- | --- | --- |
| **Crispr 1** |  |  |  |
| BBC27_RS03890 | 450 | Type III-B CRISPR module RAMP protein Cmr6 (*Tepidiphilus thermophilus*) | 54 |
| BBC27_RS03895 | 120 | Type III-B CRISPR module-associated protein Cmr5 (*Thauera butanivorans*) | 66 |
| BBC27_RS03900 | 301 | Type III-B CRISPR module RAMP protein Cmr4 (*Ferrovum* sp. Z-31) | 73 |
| BBC27_RS03905 | 394 | Type III-B CRISPR module RAMP protein Cmr3 (*Methylohalobius crimeensis*) | 48 |
| BBC27_RS03910 | 1007 | Type III-B CRISPR-associated protein Cas10/Cmr2 (*Thauera* sp. SWB20) | 67 |
| BBC27_RS03915 | 342 | CRISPR-associated protein Cmr1 (*Halorhodospira halophila* SL1) | 33 |
| BBC27_RS03930 | 384 | Putative cas protein  (*Thauera* sp. SWB20) | 33 |
| BBC27_RS03935 | 378 | CRISPR-associated protein, NE0113 family (*Allochromatium vinosum*) | 52 |
| BBC27_RS03940 | 251 | CRISPR-associated protein Cas6  (*Candidatus Magnetoglobus* multicellularis str. Araruama) | 32 |
| BBC27_RS03945 | 92 | CRISPR-associated endonuclease Cas2 (*Methylomarinum vadi*) | 52 |
| BBC27_RS03950 | 320 | CRISPR-associated endonuclease Cas1 (*Nitrosococcus halophilus*) | 43 |
| BBC27_RS03955 | 231 | CRISPR associated protein Cas1  (*Selenomonas ruminantium*) | 28 |
| BBC27_RS03960 | 113 | CRISPR-associated endoribonuclease Cas2 (*Aggregatibacter* sp. oral taxon 458) | 40 |
| **Crispr 2** |  |  |  |
| BBC27_RS08720 | 312 | subtype I-F CRISPR-associated endonuclease Cas1 (*Thiomonas* sp. FB-Cd) | 73 |
| BBC27_RS08725 | 193 | type I-F CRISPR-associated endoribonuclease Cas6/Csy4 (*Acidithiobacillus thiooxidans*) | 75 |
| BBC27_RS08730 | 349 | type I-F CRISPR-associated protein Csy3 (*Acidithiobacillus thiooxidans*) | 84 |
| BBC27_RS08735 | 271 | type I-F CRISPR-associated protein Csy2 (*Desulfonatronospira thiodismutans*) | 29 |
| BBC27_RS08750 | 874 | CRISPR-associated endonuclease Cas3’’ (*Desulfonatronospira thiodismutans*) | 33 |
