# Supplementary material for: Genomic and transcriptomic analyses reveal adaptation mechanisms of an Acidithiobacillus ferrivorans strain YL15 to alpine acid mine drainage
Source: PLoS One. 2017 May 19;12(5):e0178008. doi: 10.1371/journal.pone.0178008 (PMC5438186; doi:10.1371/journal.pone.0178008)
Supplement: S6 Table — There were 199 and 173 genes with significantly higher and lower RNA transcripts out of the total 2,798 protein-coding genes in genome of strain YL15. Classification of protein functions was based on KEGG annotation. (DOCX) [file pone.0178008.s008.docx]

**S6 Table.** **Functional genes with differential transcritpts based on analysis by TopHat and Cufflinks packages.** There were 199 and 173 genes with significantly higher and lower RNA transcripts out of the total 2,798 protein-coding genes in genome of strain YL15. Classification of protein functions was based on KEGG annotation.

| Gene | Protein | log_2_(fold  change) | Adjusted  p-value | Classification |
| --- | --- | --- | --- | --- |
| BBC27_RS00085 | Transcription termination/antitermination protein NusA | 1.5828 | 4.58E-04 | Transcription regulation |
| BBC27_RS00090 | Ribosome maturation factor | 1.73702 | 4.58E-04 | No hits |
| BBC27_RS00320 | Formate dehydrogenase | 1.41369 | 4.58E-04 | No hits |
| BBC27_RS00365 | Extradiol dioxygenase | 1.48835 | 4.84E-03 | No hits |
| BBC27_RS00600 | Inorganic pyrophosphatase | 1.30119 | 2.31E-02 | Oxidative phosphorylation |
| BBC27_RS00720 | Photosystem reaction center subunit H | 1.49415 | 9.13E-03 | No hits |
| BBC27_RS01030 | 50S ribosomal protein L19 | 1.08754 | 1.15E-03 | Ribosome |
| BBC27_RS01035 | tRNA (guanosine(37)-N1)-methyltransferase  TrmD, partial | 1.22338 | 2.05E-03 | No hits |
| BBC27_RS01100 | Biopolymer transporter ExbD | 2.8587 | 4.58E-04 | No hits |
| BBC27_RS01105 | Flagellar motor protein MotA | 2.8587 | 4.58E-04 | Motility/flagellar |
| BBC27_RS01110 | Hypothetical protein | 1.95436 | 8.19E-04 | No hits |
| BBC27_RS01370 | Ni/Fe hydrogenase | 2.32486 | 1.66E-02 | No hits |
| BBC27_RS01380 | Phosphate ABC transporter, permease protein PstA | 1.33632 | 4.58E-04 | Transmembrane transport |
| BBC27_RS01385 | Phosphate ABC transporter permease subunit PstC | 1.33632 | 4.58E-04 | Transmembrane transport |
| BBC27_RS01390 | Phosphate ABC transporter substrate-binding  protein PstS | 2.49827 | 4.58E-04 | Transmembrane transport |
| BBC27_RS01410 | Ribosome biogenesis GTPase Der | 1.14983 | 1.77E-03 | No hits |
| BBC27_RS01480 | ABC transporter | 1.72401 | 1.15E-02 | Transmembrane transport |
| BBC27_RS01650 | Secretion protein | 1.1444 | 3.30E-03 | No hits |
| BBC27_RS01655 | Deacylase | 1.18277 | 9.96E-03 | No hits |
| BBC27_RS01705 | Signal recognition particle protein | 1.24083 | 4.58E-04 | Protein export |
| BBC27_RS01710 | 30S ribosomal protein S16 | 1.14198 | 2.81E-03 | Ribosome |
| BBC27_RS01715 | 16S rRNA processing protein RimM | 1.14198 | 2.81E-03 | No hits |
| BBC27_RS01895 | Hypothetical protein | 1.11048 | 2.56E-03 | No hits |
| BBC27_RS01900 | Acetolactate synthase, large subunit, biosynthetic type | 1.1535 | 2.05E-03 | No hits |
| BBC27_RS01995 | Preprotein translocase subunit SecE | 1.54179 | 4.58E-04 | Protein export/sec pathway |
| BBC27_RS02000 | MULTISPECIES: transcription  termination/antitermination protein NusG | 1.02577 | 8.19E-04 | Transcription regulation |
| BBC27_RS02005 | 50S ribosomal protein L11 | 1.07119 | 4.58E-04 | Ribosome |
| BBC27_RS02010 | 50S ribosomal protein L1 | 1.06241 | 4.58E-04 | Ribosome |
| BBC27_RS02015 | 50S ribosomal protein L10 | 3.10729 | 4.58E-04 | Ribosome |
| BBC27_RS02025 | DNA-directed RNA polymerase subunit beta | 2.44591 | 4.58E-04 | RNA polymerase |
| BBC27_RS02030 | DNA-directed RNA polymerase subunit beta\' | 1.3579 | 4.58E-04 | RNA polymerase |
| BBC27_RS02035 | 30S ribosomal protein S12 | 2.04477 | 4.58E-04 | Ribosome |
| BBC27_RS02040 | 30S ribosomal protein S7 | 1.51739 | 4.58E-04 | Ribosome |
| BBC27_RS02045 | Translation elongation factor G | 1.98945 | 4.58E-04 | Translation regulation |
| BBC27_RS02240 | Hypothetical protein | 1.73522 | 9.74E-03 | No hits |
| BBC27_RS02275 | Flagellar motor protein MotA | 1.80892 | 4.58E-04 | Motility/flagellar |
| BBC27_RS02280 | Biopolymer transporter ExbD | 1.80892 | 4.58E-04 | No hits |
| BBC27_RS02285 | Hypothetical protein | 1.31208 | 7.19E-03 | No hits |
| BBC27_RS02290 | Hypothetical protein | 1.31208 | 7.19E-03 | No hits |
| BBC27_RS02295 | Tetraacyldisaccharide 4\'-kinase, partial | 1.31208 | 7.19E-03 | Lipopolysaccharide biosynthesis |
| BBC27_RS02725 | Membrane protein | 2.02499 | 4.58E-04 | No hits |
| BBC27_RS02890 | Radical SAM protein | 1.20963 | 4.58E-04 | No hits |
| BBC27_RS02930 | Hypothetical protein | 1.08916 | 3.10E-02 | No hits |
| BBC27_RS02975 | MULTISPECIES: 50S ribosomal protein L13 | 1.7029 | 4.58E-04 | Ribosome |
| BBC27_RS03140 | 50S ribosomal protein L17 | 1.69924 | 4.58E-04 | Ribosome |
| BBC27_RS03145 | DNA-directed RNA polymerase subunit alpha | 2.06067 | 4.58E-04 | RNA polymerase |
| BBC27_RS03150 | 30S ribosomal protein S4 | 1.74918 | 4.58E-04 | Ribosome |
| BBC27_RS03155 | 30S ribosomal protein S11 | 2.03055 | 4.58E-04 | Ribosome |
| BBC27_RS03160 | MULTISPECIES: 30S ribosomal protein S13 | 1.03839 | 1.15E-03 | Ribosome |
| BBC27_RS03165 | MULTISPECIES: translation initiation factor IF-1 | 2.34 | 4.58E-04 | Translation regulation |
| BBC27_RS03170 | Adenylate kinase | 1.15896 | 4.58E-04 | Purine metabolism |
| BBC27_RS03175 | Preprotein translocase subunit SecY | 1.15896 | 4.58E-04 | Protein export/sec pathway |
| BBC27_RS03180 | 50S ribosomal protein L15 | 1.15896 | 4.58E-04 | Ribosome |
| BBC27_RS03185 | MULTISPECIES: 50S ribosomal protein L30 | 1.15896 | 4.58E-04 | Ribosome |
| BBC27_RS03190 | 30S ribosomal protein S5 | 1.15896 | 4.58E-04 | Ribosome |
| BBC27_RS03195 | 50S ribosomal protein L18 | 1.12783 | 4.58E-04 | Ribosome |
| BBC27_RS03200 | 50S ribosomal protein L6 | 1.7877 | 4.58E-04 | Ribosome |
| BBC27_RS03205 | 30S ribosomal protein S8 | 2.06644 | 4.58E-04 | Ribosome |
| BBC27_RS03210 | 50S ribosomal protein L5 | 1.57456 | 4.58E-04 | Ribosome |
| BBC27_RS03215 | 50S ribosomal protein L24 | 1.6145 | 4.58E-04 | Ribosome |
| BBC27_RS03220 | MULTISPECIES: 50S ribosomal protein L14 | 1.24876 | 4.58E-04 | Ribosome |
| BBC27_RS03225 | MULTISPECIES: 30S ribosomal protein S17 | 1.24876 | 4.58E-04 | Ribosome |
| BBC27_RS03230 | 50S ribosomal protein L29 | 1.24876 | 4.58E-04 | Ribosome |
| BBC27_RS03235 | MULTISPECIES: 50S ribosomal protein L16 | 1.24876 | 4.58E-04 | Ribosome |
| BBC27_RS03240 | 30S ribosomal protein S3 | 2.18791 | 4.58E-04 | Ribosome |
| BBC27_RS03245 | MULTISPECIES: 50S ribosomal protein L22 | 2.01629 | 4.58E-04 | Ribosome |
| BBC27_RS03250 | MULTISPECIES: 30S ribosomal protein S19 | 1.6372 | 4.58E-04 | Ribosome |
| BBC27_RS03255 | 50S ribosomal protein L2 | 1.57415 | 4.58E-04 | Ribosome |
| BBC27_RS03260 | MULTISPECIES: 50S ribosomal protein L23 | 1.47632 | 4.58E-04 | Ribosome |
| BBC27_RS03265 | 50S ribosomal protein L4 | 1.47632 | 4.58E-04 | Ribosome |
| BBC27_RS03270 | 50S ribosomal protein L3 | 1.76373 | 4.58E-04 | Ribosome |
| BBC27_RS03275 | MULTISPECIES: 30S ribosomal protein S10 | 2.02709 | 4.58E-04 | Ribosome |
| BBC27_RS03380 | Glycerol-3-phosphate dehydrogenase | 1.37532 | 2.56E-03 | Lipid metabolism |
| BBC27_RS03395 | Hypothetical protein | 2.77654 | 4.58E-04 | No hits |
| BBC27_RS03460 | Penicillin-binding protein 2 | 1.13158 | 4.58E-04 | Peptidoglycan biosynthesis |
| BBC27_RS03465 | 50S ribosomal protein L25/general stress protein Ctc | 1.13158 | 4.58E-04 | Ribosome |
| BBC27_RS03470 | Aminoacyl-tRNA hydrolase | 1.13158 | 4.58E-04 | No hits |
| BBC27_RS03475 | GTP-binding protein YchF | 1.13158 | 4.58E-04 | No hits |
| BBC27_RS03480 | Hypothetical protein | 1.13158 | 4.58E-04 | No hits |
| BBC27_RS03485 | Hypothetical protein | 1.13158 | 4.58E-04 | No hits |
| BBC27_RS03490 | ATP-dependent exonuclease SbcCD, C subunit-like protein | 1.13158 | 4.58E-04 | No hits |
| BBC27_RS03495 | Hypothetical protein | 1.13158 | 4.58E-04 | No hits |
| BBC27_RS03500 | DNA-binding protein | 1.13158 | 4.58E-04 | No hits |
| BBC27_RS03710 | Molecular chaperone HtpG | 1.31127 | 4.58E-04 | Protein processing/chaperone |
| BBC27_RS04100 | Hypothetical protein | 1.82124 | 4.58E-04 | No hits |
| BBC27_RS04770 | Phosphoglucosamine mutase | 1.52886 | 1.15E-03 | Amino sugar and nucleotide  sugar metabolism |
| BBC27_RS04870 | Threonine--tRNA ligase | 2.20503 | 4.58E-04 | Aminoacyl-tRNA biosynthesis |
| BBC27_RS04875 | MULTISPECIES: translation initiation factor IF-3 | 1.84219 | 4.58E-04 | Translation regulation |
| BBC27_RS04880 | 50S ribosomal protein L35 | 1.14795 | 7.03E-03 | Ribosome |
| BBC27_RS04885 | MULTISPECIES: 50S ribosomal protein L20 | 1.00441 | 4.58E-04 | Ribosome |
| BBC27_RS04890 | Phenylalanine--tRNA ligase subunit alpha | 1.42599 | 4.58E-04 | Aminoacyl-tRNA biosynthesis |
| BBC27_RS04895 | Phenylalanine--tRNA ligase subunit beta | 1.00902 | 4.58E-04 | Aminoacyl-tRNA biosynthesis |
| BBC27_RS04970 | Permease | 1.10668 | 1.91E-02 | Beta-Lactam resistance |
| BBC27_RS05145 | Hypothetical protein | 2.5352 | 4.58E-04 | No hits |
| BBC27_RS05155 | Porin | 1.25858 | 4.58E-04 | No hits |
| BBC27_RS05165 | Hypothetical protein | 1.55714 | 8.90E-03 | No hits |
| BBC27_RS05505 | Thiosulfate oxidation carrier protein SoxY | 1.39498 | 2.57E-02 | Sulfur metabolism/sulfur oxidation |
| BBC27_RS05665 | ATP-binding protein | 1.60248 | 4.58E-04 | No hits |
| BBC27_RS05770 | Hypothetical protein | 1.0509 | 4.58E-04 | No hits |
| BBC27_RS05775 | Outer membrane lipoprotein LolB | 1.0509 | 4.58E-04 | No hits |
| BBC27_RS05780 | 4-(cytidine 5\'-diphospho)-2-C-methyl-D-  erythritol kinase | 1.0509 | 4.58E-04 | Terpenoid backbone biosynthesis |
| BBC27_RS05790 | Ribose-phosphate pyrophosphokinase | 1.30766 | 2.31E-03 | Carbon metabolism |
| BBC27_RS05985 | Hypothetical protein | 1.30755 | 3.06E-03 | No hits |
| BBC27_RS06075 | RNA polymerase sigma factor RpoD | 1.62147 | 4.61E-03 | RNA polymerase |
| BBC27_RS06080 | DNA primase | 1.62147 | 4.61E-03 | DNA replication |
| BBC27_RS06085 | DNA mismatch repair protein MutS | 1.62147 | 4.61E-03 | Mismatch repair |
| BBC27_RS06090 | Aspartyl-tRNA amidotransferase subunit B | 1.62147 | 4.61E-03 | No hits |
| BBC27_RS06095 | MULTISPECIES: 30S ribosomal protein S21 | 1.62147 | 4.61E-03 | Ribosome |
| BBC27_RS06235 | Toxin YoeB | 1.40865 | 1.60E-02 | No hits |
| BBC27_RS06240 | MULTISPECIES: prevent-host-death family protein | 1.40865 | 1.60E-02 | No hits |
| BBC27_RS06245 | Tetrathionate hydrolase | 1.41488 | 6.56E-03 | Sulfur metabolism/sulfur oxidation |
| BBC27_RS07205 | Cysteine desulfurase | 1.22465 | 4.58E-04 | Thiamine metabolism |
| BBC27_RS07210 | DNA-binding protein | 1.01387 | 4.58E-04 | No hits |
| BBC27_RS07270 | N utilization substance protein B | 1.82068 | 1.26E-02 | No hits |
| BBC27_RS07300 | Serine hydroxymethyltransferase | 1.41534 | 4.58E-04 | Carbon metabolism |
| BBC27_RS07535 | Hypothetical protein, partial | 1.16926 | 4.58E-04 | Fructose and mannose metabolism |
| BBC27_RS07825 | Phosphoribosylaminoimidazolesuccinocarboxamide synthase | 1.08946 | 4.58E-04 | Purine metabolism |
| BBC27_RS07830 | Adenylosuccinate lyase | 1.08946 | 4.58E-04 | Purine metabolism |
| BBC27_RS07835 | Hypothetical protein | 1.08946 | 4.58E-04 | No hits |
| BBC27_RS08210 | Hypothetical protein | 1.37969 | 1.37E-02 | No hits |
| BBC27_RS08295 | RND transporter | 1.6211 | 1.15E-03 | No hits |
| BBC27_RS08300 | Efflux transporter periplasmic adaptor subunit | 1.08748 | 4.58E-04 | No hits |
| BBC27_RS08350 | Hypothetical protein | 1.48815 | 2.31E-03 | No hits |
| BBC27_RS08355 | Peptidylprolyl isomerase | 1.20028 | 4.58E-04 | Protein processing/chaperone |
| BBC27_RS08410 | Integration host factor subunit beta | 1.16479 | 2.56E-03 | No hits |
| BBC27_RS08435 | 3-deoxy-7-phosphoheptulonate synthase | 1.46593 | 4.58E-04 | Phenylalanine, tyrosine and  tryptophan biosynthesis |
| BBC27_RS08565 | 2-isopropylmalate synthase | 1.73511 | 1.20E-02 | Biosynthesis of amino acids |
| BBC27_RS08595 | Cyclophilin | 1.25021 | 4.58E-04 | No hits |
| BBC27_RS08720 | Subtype I-F CRISPR-associated endonuclease Cas1 | 1.64053 | 8.19E-04 | No hits |
| BBC27_RS08725 | Type I-F CRISPR-associated  endoribonuclease Cas6/Csy4 | 1.64053 | 8.19E-04 | No hits |
| BBC27_RS08995 | Oxidoreductase | 2.63899 | 4.58E-04 | Oxidative phosphorylation |
| BBC27_RS09005 | Hypothetical protein | 1.82587 | 1.38E-02 | No hits |
| BBC27_RS09010 | Fructose-bisphosphate aldolase | 1.82587 | 1.38E-02 | No hits |
| BBC27_RS09015 | VWA domain-containing protein | 1.95377 | 4.58E-04 | No hits |
| BBC27_RS09020 | ATPase AAA | 2.58889 | 4.58E-04 | No hits |
| BBC27_RS09025 | Hypothetical protein | 2.85212 | 1.15E-03 | No hits |
| BBC27_RS09030 | Cobyrinic acid a,c-diamide synthase | 2.85212 | 1.15E-03 | No hits |
| BBC27_RS09035 | Hypothetical protein | 3.33273 | 1.45E-03 | No hits |
| BBC27_RS09040 | Bacterioferritin | 3.33273 | 1.45E-03 | No hits |
| BBC27_RS09560 | Protein-export membrane protein SecF | 1.49342 | 4.58E-04 | Protein export/sec pathway |
| BBC27_RS09570 | Preprotein translocase subunit YajC | 1.17246 | 3.83E-03 | Protein export/sec pathway |
| BBC27_RS10385 | Hypothetical protein | 1.01526 | 1.45E-03 | Motility/flagellar |
| BBC27_RS10415 | Hypothetical protein | 1.21126 | 1.02E-02 | Chemotaxis |
| BBC27_RS10445 | Hypothetical protein | 1.85069 | 4.58E-04 | Chemotaxis |
| BBC27_RS10455 | Hypothetical protein | 3.15374 | 4.58E-04 | No hits |
| BBC27_RS10505 | Tanscription elongation factor GreA | 1.17376 | 3.65E-02 | Transcription regulation |
| BBC27_RS10885 | Hypothetical protein | 1.61174 | 4.58E-04 | No hits |
| BBC27_RS10945 | Hypothetical protein | 1.6968 | 1.77E-03 | No hits |
| BBC27_RS10960 | Hypothetical protein | 1.25025 | 4.40E-03 | No hits |
| BBC27_RS11050 | damage-inducible protein | 1.15994 | 7.63E-03 | No hits |
| BBC27_RS11055 | phosphomannomutase | 1.33874 | 1.15E-02 | Fructose and mannose metabolism |
| BBC27_RS11060 | Hypothetical protein | 1.33874 | 1.15E-02 | No hits |
| BBC27_RS11065 | ABC-F family ATPase | 2.03772 | 4.58E-04 | No hits |
| BBC27_RS11070 | Hypothetical protein | 1.24863 | 4.58E-04 | No hits |
| BBC27_RS11075 | DNA-binding protein | 1.24863 | 4.58E-04 | No hits |
| BBC27_RS11080 | DNA methylase N-4 | 1.24863 | 4.58E-04 | No hits |
| BBC27_RS11085 | Zeta toxin family protein | 1.24863 | 4.58E-04 | No hits |
| BBC27_RS11090 | Hypothetical protein | 1.24863 | 4.58E-04 | No hits |
| BBC27_RS11100 | phosphomethylpyrimidine synthase ThiC | 1.24863 | 4.58E-04 | Thiamine metabolism |
| BBC27_RS11205 | Hypothetical protein | 1.2229 | 4.61E-03 | No hits |
| BBC27_RS11210 | Hypothetical protein | 1.28051 | 2.31E-03 | No hits |
| BBC27_RS11310 | CDP-glucose 4,6-dehydratase | 1.06652 | 8.19E-04 | Amino sugar and nucleotide  sugar metabolism |
| BBC27_RS11430 | Hypothetical protein | 1.09958 | 4.58E-04 | No hits |
| BBC27_RS11490 | LysR family transcriptional regulator | 2.44296 | 3.30E-03 | No hits |
| BBC27_RS11495 | Ribulose-bisphosphate carboxylase large subunit | 3.45966 | 4.58E-04 | Carbon metabolism |
| BBC27_RS11500 | Ribulose bisphosphate carboxylase small subunit | 2.0969 | 4.58E-04 | Carbon metabolism |
| BBC27_RS11510 | Transcriptional initiation protein Tat, partial | 1.40241 | 4.58E-04 | Transcription regulation |
| BBC27_RS11515 | Carboxysome shell carbonic anhydrase | 1.75041 | 2.31E-03 | No hits |
| BBC27_RS11520 | Carboxysome peptide A | 1.75041 | 2.31E-03 | No hits |
| BBC27_RS11590 | Transcription-repair coupling factor | 1.01248 | 4.86E-02 | Transcription regulation |
| BBC27_RS11745 | Transketolase | 2.16546 | 4.58E-04 | No hits |
| BBC27_RS11750 | Hrp-dependent type III effector protein | 1.83061 | 8.19E-04 | No hits |
| BBC27_RS11755 | Glycolate oxidase subunit GlcD | 1.53828 | 9.53E-03 | Glyoxylate and  dicarboxylate metabolism |
| BBC27_RS11760 | Glycolate oxidase subunit GlcE | 1.53828 | 9.53E-03 | Glyoxylate and  dicarboxylate metabolism |
| BBC27_RS11765 | Glycolate oxidase iron-sulfur subunit | 1.21241 | 4.72E-02 | Glyoxylate and  dicarboxylate metabolism |
| BBC27_RS11785 | Magnesium transporter | 1.01891 | 3.06E-02 | No hits |
| BBC27_RS11840 | ATP synthase F0F1 subunit A | 2.82125 | 4.58E-04 | Energy metabolism/ATP synthesis |
| BBC27_RS11845 | MULTISPECIES: ATP synthase subunit C | 1.13572 | 3.16E-02 | Energy metabolism/ATP synthesis |
| BBC27_RS11850 | ATP synthase F0 subunit B | 1.34445 | 4.58E-04 | Energy metabolism/ATP synthesis |
| BBC27_RS11855 | ATP synthase subunit delta | 1.06333 | 2.81E-03 | Energy metabolism/ATP synthesis |
| BBC27_RS11860 | F0F1 ATP synthase subunit alpha | 2.47167 | 4.58E-04 | Energy metabolism/ATP synthesis |
| BBC27_RS11865 | F0F1 ATP synthase subunit gamma | 1.74462 | 4.58E-04 | Energy metabolism/ATP synthesis |
| BBC27_RS12050 | Cold-shock protein | 1.586 | 4.58E-04 | No hits |
| BBC27_RS12090 | Twin arginine-targeting protein translocase TatB | 1.03848 | 8.19E-04 | No hits |
| BBC27_RS12620 | Ferritin | 1.12977 | 8.19E-04 | No hits |
| BBC27_RS12625 | Hypothetical protein | 1.12977 | 8.19E-04 | No hits |
| BBC27_RS12980 | Metal transporter | 1.31309 | 4.58E-04 | No hits |
| BBC27_RS12985 | Hypothetical protein | 1.31309 | 4.58E-04 | No hits |
| BBC27_RS13035 | Aspartate--tRNA ligase | 1.26847 | 4.58E-04 | Aminoacyl-tRNA biosynthesis |
| BBC27_RS13050 | Transcriptional regulator | 1.3587 | 1.77E-03 | No hits |
| BBC27_RS13560 | Hypothetical protein | 1.91323 | 4.58E-04 | No hits |
| BBC27_RS13565 | Cytochrome C | 1.57865 | 4.58E-04 | Energy metabolism/electron transfer |
| BBC27_RS13570 | Short-chain dehydrogenase | 1.57865 | 4.58E-04 | Iron oxidation |
| BBC27_RS14625 | ATP-dependent RNA helicase RhlE | 1.88808 | 2.05E-03 | RNA degradation |
| BBC27_RS14775 | 50S ribosomal protein L9 | 1.29165 | 4.58E-04 | Ribosome |
| BBC27_RS14780 | Hypothetical protein, partial | 2.56365 | 4.58E-04 | No hits |
| BBC27_RS14785 | Elongation factor Tu | 1.77274 | 4.58E-04 | Translation regulation |
| BBC27_RS14870 | Malate dehydrogenase, partial | 1.23955 | 1.45E-03 | Carbon metabolism |
| BBC27_RS14960 | Hypothetical protein | 1.11567 | 5.34E-03 | No hits |
| BBC27_RS00700 | NYN domain-containing protein | -1.28097 | 4.58E-04 | No hits |
| BBC27_RS00755 | Dihydroorotate dehydrogenase | -1.52672 | 4.58E-04 | Terpenoid backbone biosynthesis |
| BBC27_RS00970 | Hypothetical protein | -2.28557 | 1.94E-02 | No hits |
| BBC27_RS01050 | Hypothetical protein | -1.25545 | 2.68E-02 | No hits |
| BBC27_RS01130 | Hypothetical protein | -1.6082 | 4.58E-04 | Two-component system |
| BBC27_RS01185 | Trehalose synthase | -2.12653 | 4.58E-04 | Starch and sucrose metabolism |
| BBC27_RS01190 | Glycogen debranching enzyme GlgX | -1.93626 | 7.19E-03 | Starch and sucrose metabolism |
| BBC27_RS01195 | Malto-oligosyltrehalose trehalohydrolase | -1.93626 | 7.19E-03 | Starch and sucrose metabolism |
| BBC27_RS01495 | Apolipoprotein N-acyltransferase | -1.62534 | 2.31E-03 | No hits |
| BBC27_RS01500 | Magnesium/cobalt efflux protein | -1.62534 | 2.31E-03 | No hits |
| BBC27_RS01505 | rRNA maturation RNase YbeY | -1.62534 | 2.31E-03 | No hits |
| BBC27_RS01510 | Phosphate starvation protein PhoH | -1.62534 | 2.31E-03 | No hits |
| BBC27_RS01515 | tRNA (N6-isopentenyl adenosine(37)-C2)-  methylthiotransferase MiaB | -1.62534 | 2.31E-03 | No hits |
| BBC27_RS01520 | Hypothetical protein | -1.62534 | 2.31E-03 | No hits |
| BBC27_RS01620 | Phosphatase | -1.49459 | 4.58E-04 | No hits |
| BBC27_RS01625 | AI-2E family transporter | -1.49459 | 4.58E-04 | No hits |
| BBC27_RS01630 | Hypothetical protein | -1.49459 | 4.58E-04 | No hits |
| BBC27_RS01635 | Hypothetical protein | -1.49459 | 4.58E-04 | No hits |
| BBC27_RS01640 | 23S rRNA (guanosine(2251)-2\'-O)-  methyltransferase RlmB, partial | -1.49459 | 4.58E-04 | No hits |
| BBC27_RS01750 | Hlutamine amidotransferase | -1.71387 | 4.58E-04 | No hits |
| BBC27_RS01755 | Ribose 5-phosphate isomerase A | -1.71387 | 4.58E-04 | Carbon metabolism |
| BBC27_RS01760 | Pyridoxamine 5\'-phosphate oxidase | -1.71387 | 4.58E-04 | Vitamin B6 metabolism |
| BBC27_RS01905 | MULTISPECIES: prevent-host-death protein | -1.01393 | 3.55E-03 | No hits |
| BBC27_RS01935 | Hypothetical protein | -1.15874 | 2.79E-02 | No hits |
| BBC27_RS01940 | Cytochrome o ubiquinol oxidase | -1.15874 | 2.79E-02 | Energy metabolism/electron transfer |
| BBC27_RS01945 | Cytochrome o ubiquinol oxidase subunit I | -1.24975 | 4.58E-04 | Energy metabolism/electron transfer |
|  | Cytochrome o ubiquinol oxidase | -1.53854 | 2.07E-02 | Energy metabolism/electron transfer |
| BBC27_RS01955 | Hypothetical protein | -1.67801 | 4.58E-04 | No hits |
| BBC27_RS01960 | Pyridine nucleotide-disulfide oxidoreductase | -2.04408 | 4.58E-04 | No hits |
| BBC27_RS02170 | K^+^-transporting ATPase subunit F | -7.86209 | 2.05E-03 | No hits |
| BBC27_RS02175 | Potassium-transporting ATPase subunit KdpA | -7.86209 | 2.05E-03 | No hits |
| BBC27_RS02355 | Transposase | -1.42952 | 1.45E-03 | No hits |
| BBC27_RS02805 | Phage shock protein A | -1.08173 | 4.58E-04 | No hits |
| BBC27_RS02810 | Hypothetical protein | -1.22309 | 4.07E-02 | No hits |
| BBC27_RS02830 | Hypothetical protein | -1.35901 | 4.58E-04 | No hits |
| BBC27_RS02910 | Hypothetical protein | -1.15055 | 4.58E-04 | No hits |
| BBC27_RS03055 | Dihydroorotase | -1.36226 | 8.03E-03 | Pyrimidine metabolism |
| BBC27_RS03060 | Hypothetical protein | -1.45283 | 4.58E-04 | No hits |
| BBC27_RS03065 | Hypothetical protein | -2.00114 | 1.15E-03 | No hits |
| BBC27_RS03405 | Universal stress protein | -1.42665 | 4.58E-04 | No hits |
| BBC27_RS03690 | Hypothetical protein | -1.93861 | 3.66E-02 | No hits |
| BBC27_RS03695 | Diguanylate cyclase | -1.93861 | 3.66E-02 | No hits |
| BBC27_RS04110 | Exodeoxyribonuclease V subunit alpha | -1.59229 | 1.18E-02 | Homologous recombination |
| BBC27_RS04115 | Exodeoxyribonuclease V subunit beta, partial | -1.59229 | 1.18E-02 | Homologous recombination |
| BBC27_RS04210 | Amidase | -2.15531 | 1.58E-02 | No hits |
| BBC27_RS04215 | Hypothetical protein | -2.15531 | 1.58E-02 | No hits |
| BBC27_RS04225 | Amidohydrolase | -1.74443 | 8.20E-03 | No hits |
| BBC27_RS04310 | Phosphoenolpyruvate synthase | -1.84472 | 4.58E-04 | Carbon metabolism |
| BBC27_RS04315 | Phosphoglycerate mutase  (2,3-diphosphoglycerate-independent) | -1.84472 | 4.58E-04 | Carbon metabolism |
| BBC27_RS04320 | 2-oxo acid dehydrogenase | -1.36452 | 4.58E-04 | Carbon metabolism |
| BBC27_RS04325 | Acetate kinase | -1.36452 | 4.58E-04 | Carbon metabolism |
| BBC27_RS04330 | MFS transporter | -1.36452 | 4.58E-04 | No hits |
| BBC27_RS04350 | Integration host factor subunit alpha | -1.7455 | 4.58E-04 | No hits |
| BBC27_RS04360 | RNA helicase | -1.2943 | 8.19E-04 | No hits |
| BBC27_RS04365 | Type III restriction endonuclease subunit M | -1.33417 | 4.07E-02 | No hits |
| BBC27_RS04425 | Hypothetical protein | -2.03482 | 1.03E-02 | No hits |
| BBC27_RS04510 | Apolipoprotein acyltransferase | -1.64943 | 4.07E-02 | No hits |
| BBC27_RS04515 | Hypothetical protein | -1.64943 | 4.07E-02 | No hits |
| BBC27_RS04570 | Plasma-membrane proton-efflux P-type ATPase | -1.46489 | 2.56E-03 | No hits |
| BBC27_RS04595 | DNA polymerase I | -1.17561 | 2.32E-02 | DNA replication |
| BBC27_RS04625 | Cytochrome C biogenesis protein | -2.04996 | 4.58E-04 | No hits |
| BBC27_RS04630 | MerR family transcriptional regulator | -2.04996 | 4.58E-04 | No hits |
| BBC27_RS04635 | Phosphoribosyl transferase | -2.04996 | 4.58E-04 | No hits |
| BBC27_RS04725 | PbsX family transcriptional regulator | -2.69956 | 4.58E-04 | No hits |
| BBC27_RS04730 | DNA-binding protein | -2.69956 | 4.58E-04 | No hits |
| BBC27_RS05520 | Two-component sensor histidine kinase | -1.59573 | 2.06E-02 | Two-component system |
| BBC27_RS05625 | Oxidoreductase | -1.76913 | 1.62E-02 | Aminobenzoate degradation |
| BBC27_RS05705 | Hypothetical protein | -1.27909 | 1.45E-03 | No hits |
| BBC27_RS05710 | RNA polymerase factor sigma-32 | -1.27909 | 1.45E-03 | RNA polymerase |
| BBC27_RS05945 | Hypothetical protein | -1.13881 | 9.34E-03 | No hits |
| BBC27_RS05960 | Hypothetical protein | -2.09381 | 1.15E-03 | No hits |
| BBC27_RS06130 | Ferritin | -1.38825 | 4.58E-04 | No hits |
| BBC27_RS06340 | Porin | -1.73228 | 1.09E-02 | No hits |
| BBC27_RS06520 | 2-nitropropane dioxygenase | -1.4072 | 2.31E-02 | Nitrogen metabolism |
| BBC27_RS06525 | Lipid carrier-like protein | -2.93771 | 2.89E-02 | No hits |
| BBC27_RS06530 | Protease | -1.43818 | 1.96E-02 | No hits |
| BBC27_RS06670 | Hypothetical protein | -1.41639 | 3.89E-02 | No hits |
| BBC27_RS06675 | Hypothetical protein | -1.65719 | 4.58E-04 | No hits |
| BBC27_RS06690 | Hypothetical protein | -2.02258 | 7.40E-03 | No hits |
| BBC27_RS06800 | Hypothetical protein | -1.03026 | 8.19E-04 | No hits |
| BBC27_RS06805 | Hypothetical protein | -1.03026 | 8.19E-04 | No hits |
| BBC27_RS07050 | ABC transporter ATP-binding protein | -1.27005 | 2.79E-02 | No hits |
| BBC27_RS07055 | ABC transporter permease | -1.27005 | 2.79E-02 | No hits |
| BBC27_RS07060 | Carotenoid 1,2-hydratase | -1.27005 | 2.79E-02 | No hits |
| BBC27_RS07065 | Hypothetical protein | -1.27005 | 2.79E-02 | No hits |
| BBC27_RS07070 | Molybdopterin biosynthesis protein MoeB | -1.27005 | 2.79E-02 | Sulfur relay system |
| BBC27_RS07075 | Hypothetical protein | -1.27005 | 2.79E-02 | No hits |
| BBC27_RS07080 | Endonuclease | -1.27005 | 2.79E-02 | No hits |
| BBC27_RS07230 | Hypothetical protein | -1.37092 | 4.31E-02 | No hits |
| BBC27_RS07235 | HAD family hydrolase | -1.37092 | 4.31E-02 | Glyoxylate and  dicarboxylate metabolism |
| BBC27_RS07240 | 23S rRNA pseudouridine(955/2504/2580) synthase | -1.37092 | 4.31E-02 | No hits |
| BBC27_RS07515 | Hypothetical protein | -1.64542 | 2.17E-02 | No hits |
| BBC27_RS07980 | Hypothetical protein | -2.09929 | 1.15E-03 | No hits |
| BBC27_RS07985 | Hypothetical protein | -2.09929 | 1.15E-03 | No hits |
| BBC27_RS07990 | Hypothetical protein | -2.09929 | 1.15E-03 | No hits |
| BBC27_RS08280 | Hypothetical protein | -1.17223 | 1.28E-02 | No hits |
| BBC27_RS08310 | Bifunctional methylenetetrahydrofolate  dehydrogenase/methenyltetrahydrofolate cyclohydrolase | -1.35085 | 4.58E-04 | Carbon metabolism |
| BBC27_RS08315 | RNA degradosome polyphosphate kinase | -1.35085 | 4.58E-04 | RNA degradation |
| BBC27_RS08835 | Hypothetical protein | -1.45686 | 8.19E-04 | No hits |
| BBC27_RS09050 | Hydrogenase 4 subunit B, partial | -1.94821 | 2.84E-02 | No hits |
| BBC27_RS09055 | Formate hydrogenlyase | -1.94821 | 2.84E-02 | No hits |
| BBC27_RS09070 | Hydrogenase | -1.62155 | 4.16E-02 | No hits |
| BBC27_RS09520 | Amino acid dehydrogenase | -1.59818 | 3.15E-02 | Phenylalanine metabolism |
| BBC27_RS09660 | Molybdenum cofactor guanylyltransferase | -1.66635 | 6.81E-03 | No hits |
| BBC27_RS09665 | Molybdopterin-guanine dinucleotide  biosynthesis protein B | -1.66635 | 6.81E-03 | No hits |
| BBC27_RS09670 | Molybdopterin molybdenumtransferase MoeA | -1.66635 | 6.81E-03 | No hits |
| BBC27_RS09680 | Zn-dependent hydrolase | -1.57447 | 6.09E-03 | Sulfur metabolism |
| BBC27_RS09715 | Hypothetical protein | -1.97929 | 1.75E-02 | No hits |
| BBC27_RS10140 | Hypothetical protein, partial | -2.18186 | 4.58E-04 | No hits |
| BBC27_RS10545 | Pyridine nucleotide-disulfide oxidoreductase | -2.08916 | 2.78E-02 | No hits |
| BBC27_RS10550 | Aldolase | -2.08916 | 2.78E-02 | Carbon metabolism |
| BBC27_RS10555 | Hydroxyacid dehydrogenase | -2.08916 | 2.78E-02 | Pyruvate metabolism |
| BBC27_RS10560 | Alpha-glucan phosphorylase | -2.08916 | 2.78E-02 | Starch and sucrose metabolism |
| BBC27_RS10910 | Conjugal transfer TraD family protein | -1.0858 | 3.55E-03 | No hits |
| BBC27_RS10915 | Hypothetical protein | -2.29533 | 4.58E-04 | No hits |
| BBC27_RS11005 | Thiamine-phosphate kinase | -1.13325 | 6.56E-03 | Thiamine metabolism |
| BBC27_RS11010 | Phosphatidylglycerophosphatase | -1.13325 | 6.56E-03 | Glycerophospholipid metabolism |
| BBC27_RS11015 | Damage-inducible protein CinA | -1.13325 | 6.56E-03 | Nicotinate and  nicotinamide metabolism |
| BBC27_RS11165 | Molybdopterin synthase sulfur carrier subunit | -1.65583 | 4.07E-02 | Folate biosynthesis |
| BBC27_RS11170 | Molybdopterin converting factor | -1.65583 | 4.07E-02 | Folate biosynthesis |
| BBC27_RS11175 | YajQ family cyclic di-GMP-binding protein | -1.65583 | 4.07E-02 | No hits |
| BBC27_RS11180 | Glycine cleavage system protein T | -1.65583 | 4.07E-02 | Glycine, serine and  threonine metabolism |
| BBC27_RS11185 | Glycine cleavage system protein H | -1.65583 | 4.07E-02 | Glycine, serine and  threonine metabolism |
| BBC27_RS11190 | Glycine dehydrogenase (aminomethyl-transferring) | -1.65583 | 4.07E-02 | Glycine, serine and  threonine metabolism |
| BBC27_RS11450 | Transposase | -1.16108 | 8.19E-04 | No hits |
| BBC27_RS11555 | Hypothetical protein | -1.00979 | 3.30E-03 | No hits |
| BBC27_RS11795 | Molybdenum cofactor biosynthesis protein B | -1.60747 | 2.47E-02 | No hits |
| BBC27_RS12230 | PTS sugar transporter | -1.37691 | 4.58E-04 | Fructose and mannose metabolism |
| BBC27_RS12375 | Sporulation protein | -2.09297 | 3.30E-03 | No hits |
| BBC27_RS12380 | Arginine--tRNA ligase | -2.09297 | 3.30E-03 | Aminoacyl-tRNA biosynthesis |
| BBC27_RS12385 | Hypothetical protein | -2.09297 | 3.30E-03 | No hits |
| BBC27_RS12395 | Ribosomal protein L3 N(5)-glutamine methyltransferase | -2.09297 | 3.30E-03 | No hits |
| BBC27_RS12400 | Exodeoxyribonuclease III | -2.09297 | 3.30E-03 | Base excision repair |
| BBC27_RS12405 | Hypothetical protein | -2.09297 | 3.30E-03 | No hits |
| BBC27_RS12410 | Hypothetical protein | -2.09297 | 3.30E-03 | No hits |
| BBC27_RS12715 | Poly(A) polymerase | -2.39498 | 4.58E-04 | No hits |
| BBC27_RS13195 | Formate hydrogenlyase | -1.13074 | 2.81E-03 | No hits |
| BBC27_RS13285 | Starch synthase | -1.91556 | 4.58E-04 | Starch and sucrose metabolism |
| BBC27_RS13590 | Hypothetical protein | -1.05691 | 4.58E-04 | Starch and sucrose metabolism |
| BBC27_RS13605 | Transcriptional regulator | -1.3625 | 4.84E-03 | No hits |
| BBC27_RS13675 | Ammonium transporter | -1.08354 | 2.56E-03 | No hits |
| BBC27_RS13705 | SpoVR family protein | -2.30548 | 4.58E-04 | No hits |
| BBC27_RS13710 | Hypothetical protein | -2.30548 | 4.58E-04 | No hits |
| BBC27_RS13720 | PrkA family serine protein kinase | -2.15971 | 4.58E-04 | No hits |
| BBC27_RS14085 | Glucose dehydrogenase | -1.09713 | 8.19E-04 | Carbon metabolism |
| BBC27_RS14090 | Glycosyl hydrolase | -1.09713 | 8.19E-04 | Carbon metabolism |
| BBC27_RS14095 | Phosphoglycerate mutase | -1.37537 | 4.84E-03 | Carbon metabolism |
| BBC27_RS14100 | NADH-quinone oxidoreductase subunit N | -2.34118 | 4.58E-04 | Oxidative phosphorylation |
| BBC27_RS14105 | NADH dehydrogenase | -2.34118 | 4.58E-04 | Oxidative phosphorylation |
| BBC27_RS14110 | NADH-quinone oxidoreductase subunit L | -2.34118 | 4.58E-04 | Oxidative phosphorylation |
| BBC27_RS14120 | NADH dehydrogenase | -2.21936 | 8.43E-03 | Oxidative phosphorylation |
| BBC27_RS14125 | NADH-quinone oxidoreductase subunit I | -1.83609 | 4.58E-04 | Oxidative phosphorylation |
| BBC27_RS14130 | NADH-quinone oxidoreductase subunit H | -2.09059 | 4.58E-04 | Oxidative phosphorylation |
| BBC27_RS14135 | Hypothetical protein | -2.09059 | 4.58E-04 | No hits |
| BBC27_RS14140 | NADH dehydrogenase (quinone) subunit G, partial | -1.95583 | 4.58E-04 | Oxidative phosphorylation |
| BBC27_RS14145 | NADH-quinone oxidoreductase subunit F | -1.65866 | 4.58E-04 | Oxidative phosphorylation |
| BBC27_RS14150 | NADH-quinone oxidoreductase subunit E | -1.7339 | 2.56E-03 | Oxidative phosphorylation |
| BBC27_RS14155 | NADH-quinone oxidoreductase subunit C/D | -1.4076 | 4.58E-04 | Oxidative phosphorylation |
| BBC27_RS14170 | Hypothetical protein | -2.25734 | 5.34E-03 | No hits |
| BBC27_RS14175 | ROK family protein | -2.25734 | 5.34E-03 | Carbon metabolism |
| BBC27_RS14180 | Glucoamylase | -2.05215 | 4.58E-04 | No hits |
| BBC27_RS14185 | Glucose-6-phosphate dehydrogenase | -2.05215 | 4.58E-04 | Carbon metabolism |
| BBC27_RS14190 | 6-phosphogluconate dehydrogenase (decarboxylating) | -1.35133 | 4.29E-02 | Carbon metabolism |
| BBC27_RS14195 | Transketolase | -2.80048 | 4.58E-04 | Carbon metabolism |
| BBC27_RS14205 | Hypothetical protein | -3.67997 | 2.81E-03 | No hits |
| BBC27_RS14215 | Hypothetical protein | -1.94118 | 3.30E-03 | No hits |
| BBC27_RS14220 | Metallophosphatase | -1.94118 | 3.30E-03 | No hits |
| BBC27_RS14225 | Hypothetical protein | -1.94118 | 3.30E-03 | No hits |
| BBC27_RS14285 | Cytochrome C oxidase subunit IV | -1.24351 | 4.58E-04 | No hits |
| BBC27_RS14310 | Cytochrome C552 | -1.23583 | 4.58E-04 | No hits |
| BBC27_RS14935 | Hypothetical protein | -2.83099 | 4.58E-04 | No hits |
| BBC27_RS15135 | Hypothetical protein | -1.76866 | 1.57E-02 | No hits |
| BBC27_RS15145 | Uroporphyrinogen-III C-methyltransferase | -1.46438 | 4.72E-02 | Porphyrin and  chlorophyll metabolism |
