# Supplementary material for: Genomic and transcriptomic analyses reveal adaptation mechanisms of an Acidithiobacillus ferrivorans strain YL15 to alpine acid mine drainage
Source: PLoS One. 2017 May 19;12(5):e0178008. doi: 10.1371/journal.pone.0178008 (PMC5438186; doi:10.1371/journal.pone.0178008)
Supplement: S7 Table — The bold and regular letters represent categories and sub-categories, respectively. Sub-categories were based on classification of protein functions as listed in S6 Table. (DOCX) [file pone.0178008.s009.docx]

**S7 Table. Comparison of number of genes in main categories with higher transcripts levels at low temperatures between results from Christel et al. and this study.** The bold and regular letters represent categories and sub-categories, respectively. Sub-categories were based on classification of protein functions as listed in S6 Table.

| Category | Number of genes with higher transcript counts  at low temperatures | |
| --- | --- | --- |
|  | This study | Results from Christel et al. |
| **Transcription** |  |  |
| RNA polymerase complex | 4 | 4 |
| Transcriptional regulation | 5 | 1 |
| **Translation** |  |  |
| Ribosomal proteins | 37 | 27 |
| Translational regulation | 4 | 5 |
| **Chaperones** | 2 | 0 |
| **Transmembrane transport** |  |  |
| ABC transporters | 4 | 3 |
| Sec pathway | 4 | 2 |
| **Energy metabolism** |  |  |
| Iron oxidation | 1 | 1 |
| Sulfur oxidation | 2 | 8 |
| Electron transfer | 1 | 20 |
| ATP synthesis | 6 | 0 |
| **Chemotaxis and motility** | 5 | 0 |
| **Biofilm formation** | 0 | 6 |
| **DNA repair** | 1 | 1 |
